# Co-design of a natural fiber-timber hybrid structural system using dual-robot coreless filament winding

**DOI:** 10.1038/s41598-026-40584-6

**Published:** 2026-03-03

**Authors:** Rebeca Duque Estrada, Fabian Kannenberg, Tzu-Ying Chen, Yanan Guo, Jan Knippers, Achim Menges

**Affiliations:** 1 Institute for Computational Design and Construction (ICD), University of Stuttgart, 70174 Stuttgart, Germany; 2 Institute of Building Structures and Structural Design (ITKE), University of Stuttgart, 70174 Stuttgart, Germany; 3 Cluster of Excellence Integrative Computational Design and Construction for Architecture (IntCDC), University of Stuttgart, 70174 Stuttgart, Germany

**Keywords:** Engineering, Materials science

## Abstract

This paper presents the co-design methods for a new hybrid load-bearing system as a strategy for advancing bio-based architecture. Timber and natural fiber polymer composites (NFPC) are combined into a hybrid system, offering opportunities to leverage their strengths while balancing the use of natural resources. The system performs synergistically, with each material fulfilling complementary roles. Timber extrapolates its structural function by acting as an embedded frame for the fibers to be wound on. The paper presents computational methods designed to optimize material performance while integrating functionalities and fabrication opportunities. A dual-robot winding method is presented as a solution for balancing winding tension in the structure during fabrication. The hybrid system is demonstrated through the design and construction of a pavilion, the first to combine flax fibers with a partially bio-based resin and timber into a dual-robotically fabricated structure on an architectural scale. The project represents further advancements in multi-robot fabrication and a novel material approach toward bio-based hybrid systems in architecture.

## Introduction

 As economies grow and living standards rise, the demand for raw materials in construction is expected to nearly double by 2060^1^, further intensifying the sector’s already significant environmental impact. To reverse this trend, the construction sector must move away from petroleum-based, energy-intensive, and non-renewable materials. Instead, it must adopt strategies that lower resource consumption (dematerialization) and minimize carbon emissions (decarbonization).

Dematerialization is a strategy focused on reducing the amount of resources required for construction^2^. One of the many advantages of building with less is the reduction of emissions associated with the extraction, manufacturing, and transportation of building materials, as well as the reduction of new material extraction^1^. Additionally, with this measure, waste generation also decreases. When integrated early in the design process, dematerialization serves as a key optimization criterion, enabling the creation of performative and lightweight structures. Beyond improving material efficiency, this approach can also simplify construction, facilitating reuse and recycling, while reinforcing circular economy principles.

On the other hand, decarbonization examines the impact of these materials, addressing both embodied carbon, arising from extraction and processing, and operational carbon, generated throughout a building’s lifespan^3^. As highlighted in^1^, one pathway to achieving decarbonization and a net-zero built environment by 2060 is the responsible adoption of bio-based building materials. Bio-based materials derived from plant or animal biomass are often sourced from agricultural or forestry industries^4^ and offer a lower environmental footprint than conventional, energy-intensive alternatives. Their adoption, however, must be carefully managed within a biodiversity framework to prevent ecological imbalances. While transitioning to renewable materials is essential, excessive reliance on a single resource could threaten biodiversity and disrupt ecosystems^5^. To address these challenges, architects must move beyond simply reducing carbon footprint and adopt more comprehensive approaches that consider the broader ecological and social systems in which building materials exist, fostering ecological regeneration.

Throughout history, timber has served as a fundamental structural material in architecture and construction across various civilizations due to its accessibility, ease of processing, and lightweight properties^6^. Its practicality has made it a primary choice for construction, offering a balance between simplicity and effectiveness. In modern times, advancements in engineered timber products have significantly transformed the perception and application of this material. The introduction of engineered wood has eliminated the constraint of cross-sectional dimensions being limited by trunk size, thereby expanding the potential scale of timber structures^7^. This innovation has been particularly significant in addressing the increasing demand for renewable construction materials. With the global urban population projected to rise substantially, the newly built infrastructure by the middle of this century is expected to surpass that constructed since the onset of industrialization^8^. Consequently, timber has emerged as one of the most competitive contemporary building materials, offering a viable solution to the challenges of sustainability, climate impact, and population growth.

Studies indicate that if 90% of new urban housing were to be built with timber by 2100, global forest plantation areas would need to expand by 200% compared to current levels^9^. However, the increasing demand for timber as a primary construction material presents several challenges related to sustainability and resource management. One major concern is the risk of overexploitation, which can lead to deforestation and habitat loss. Furthermore, the timber construction industry predominantly relies on fewer than five species^10^, heightening the risk of ecosystem disruption and increasing susceptibility to insect infestations and pest outbreaks. To ensure a sustainable and resilient reliance on this natural resource, it is essential to diversify timber sources by selecting a broader range of species and expanding their geographical distribution. Additionally, incorporating other natural materials, such as natural fibers, can ease pressure on forests and the timber supply chain while fostering a more balanced approach to material sourcing.

The use of fiber polymer composites (FPC) as structural materials has been developed to replace metallic materials in the automotive and aerospace industries due to their light weight, higher specific strength and stiffness, and design flexibility^11^. With the increasing focus on sustainability, natural fiber polymer composites (NFPC) have gained research interest as an alternative reinforcement for polymers. Although natural fibers exhibit lower mechanical performance compared to synthetic fibers, their advantages, including abundance, renewability, low cost, and carbon footprint, make them an attractive subject of study^12^. One key benefit of natural fibers, such as flax, is their rapid growth cycle. While managed forests require more than 60 years before they are ready for harvest^13^, flax can be cultivated and harvested within approximately 100 days^14^. This not only enables shorter material production cycles but also provides greater flexibility in land use, allowing for adjustments based on annual market demand. In contrast, once planted, forested land remains committed to timber production for several decades.

Building on these considerations, this research aims to develop strategies for diversifying the use of natural materials by integrating NFPC and structural timber into a hybrid system. The study focuses on allocating the material roles based on their inherent properties to advance a new, performance-driven architectural hybrid morphology informed by resource potential and structural efficiency. While the composite system is not yet fully biogenic, a partially bio-based epoxy resin (39.2% bio-based content when including the hardener) is used for the first time at an architectural scale, representing a transitional step towards fully bio-based NFPC.

The concept of hybridity spans a broad spectrum of meanings, requiring a multidisciplinary approach for a comprehensive understanding^15,16^. Examining its interpretations across disciplines provides an opportunity to refine and expand the term. Within architecture, hybridity is closely linked to material science and engineering, encompassing various definitions and scales. In this context, the terms *“hybrid” and “composite”* are often used interchangeably; however, a clear distinction between these concepts is essential. Composites can be seen as a type of hybrid formed by chemically and physically distinct materials bonded to create a new material with its own thermomechanical properties^17,18^. In contrast, hybrids maintain the separability of their constituent materials, allowing each to retain its individual properties.

A theoretical investigation led by the authors^19^ explored hybridity at the intersection of material science and engineering, drawing from the frameworks of Michael Ashby^17^ and Heino Engel^20^ to develop a new perspective on hybrids in architecture. Ashby defines hybrids as a means to expand the map of material properties, achieving characteristics that no single material can provide alone^17^. Engel, in contrast, conceptualizes hybrids at a macro scale, where different structural systems are combined and share equal load-bearing responsibilities^20^. In this project, hybridity is positioned within the Architecture, Engineering, and Construction (AEC) domain as the integration of different materials into a single load-bearing system. These materials should exhibit a high degree of interdependence, leveraging complementary properties to enhance structural performance while distributing functional roles across different domains.

Building on this theoretical foundation, the project translates hybridity into a tangible structure. It represents an effort to materialize a hybrid system that embodies the definition outlined above, expanding the possibilities of hybrid tectonics while embracing the key opportunities it offers as a path to sustainable bio-based architecture.

## State of the art

The methods developed in this research contribute to and build upon the ongoing advancements in the field of hybrid systems in architecture. Simultaneously, they expand the fields of Coreless Filament Winding (CFW) and robotic fabrication by extending the use of timber beyond structural applications, integrating it as an embedded frame, and by developing and implementing a parallel dual-robot winding setup. The following sections examine state-of-the-art projects in these fields, situating this research within the broader context and highlighting its contributions.

### Hybrid systems in construction

The act of combining materials to improve certain aspects of construction has existed since the beginning of human civilization^21^. Looking at advancements driven by new computational design methods and robotic fabrication, recent research on hybrid structures highlights the potential of strategically integrating bio-based materials through digital fabrication to maximize their strengths and mitigate their weaknesses^22–25^. Research specifically on FPC-timber hybrid structures has also advanced over the last years, leveraging the distinct functional advantages of each material^26–28^.

In the Maison Fibre Pavilion^26^, FPC structures were integrated with laminated veneer lumber (LVL) plates to create walkable surfaces that effectively transfer loads into the fiber structure. The combination of high-performance carbon and glass fibers with engineered timber facilitated the development of a multi-story, lightweight building system, demonstrating the potential of CFW structures as inhabitable spaces. Building on this research, the Living Prototypes project^27^ advanced the hybrid concept by developing a preliminary slab design that strategically positioned NFPC primarily under tensile stress while subjecting timber elements to compressive forces. This project also incorporated natural flax fibers and a partially bio-based resin, demonstrating a structurally efficient morphology for a point-supported, free-plan slab system. Both projects represent an initial exploration of combining FPC and timber into a coherent hybrid system. However, they present limited material interdependencies and structural responsibilities.

The Hybrid Flax Pavilion^28^, completed in 2024, advanced this concept further by incorporating an 8-meter-span NFPC-timber hybrid beam. Cross-laminated timber (CLT) formed the top surface, bearing compressive forces while also serving as the building’s enclosure. Meanwhile, NFPC was shaped into a free-form fish-belly beam, forming a bottom chord that efficiently transfers tensile forces to the supports with variable structural depths. Through refined detailing and structural prototyping, this pavilion became the first permanent structure to integrate load-bearing NFPC-timber hybrid components.

Combining two or more materials into a cohesive structural system inherently entails a higher level of complexity, as it requires balancing factors from distinct domains such as mechanical behaviors, fabrication constraints, and environmental considerations. Co-design methods offer a way to navigate this complexity by enabling interdisciplinary negotiation between these domains^29,30^. In this context, research has emphasized the role of integrated strategies, such as a combination of virtual and physical prototyping, in advancing fibrous material systems with tailored structural and functional properties^28,31–33^. These methods support the development of performative fiber-based composite structures, allowing for more informed design decisions and efficient material use.

Expanding on these developments, the present research proposes a co-design methodology that brings together natural fiber composites and timber within a digitally driven fabrication workflow. It explores new design morphologies that further define material roles within hybrid systems, aiming to enhance both structural performance and fabrication strategies.

### Coreless filament winding

Continuous advancements in composite materials have driven the industry toward the development of lightweight and optimized fiber-reinforced components. Various manufacturing techniques have addressed a broad spectrum of industrial demands through innovative processes, products, and materials. Over the past decade, one such method, Filament Winding (FW), commonly used in the aerospace and automotive industries for producing, e.g., pressure vessels, has led to the development of CFW. The main difference between these methods lies in how the final geometry is formed. In FW, continuous fibers impregnated with a resin system are wound around a mold or mandrel to produce a predefined shape^34^, whereas in CFW, fibers are wound around anchor points located on a temporary frame, allowing the final geometry to emerge through material interaction during the winding process^35^. This shift gives the material an active role in form generation, fostering emergent behavior and geometrical freedom rather than adhering to a predetermined shape^36^. It also avoids waste and minimizes costs related to single-use molds.

Since its introduction in 2012 by Knippers et al.^37^ as a method for creating lightweight, optimized load-bearing structures in architecture, the CFW method has evolved significantly. The temporary frame, typically made of steel profiles and plates, has also advanced. Some projects^31,38–40^ adopted a modular strategy, using a consistent frame while adjusting or adding specific parts to produce varied outcomes. Others, such as the Elytra Pavilion^41^, achieved geometric variation for bespoke components by altering only the fiber arrangement within the same frame. While these earlier approaches were limited to anticlastic geometries, recent advancements, as seen in the Hybrid Flax Pavilion^28^, introduce a selectively-supported filament winding (SFW) method, which combines localized fiber support with a standard CFW frame, enabling the formation of both anticlastic and synclastic surfaces within a single component.

Compared to the single-use mandrels and molds typical of FW, the temporary frames in CFW represent an advancement in reducing both waste and costs. While portions of these frames can be repurposed after project completion, their project-specific geometries limit the potential for reuse. Different approaches, such as Spatial Winding^42^ and Spatial Lacing^43^, aim to further simplify the frame by introducing new winding logics that enable the creation of geometries independent of boundary conditions. Spatial Winding introduces a new logic where complex spatial structures emerge from just a few anchor points, achieved by guiding the fiber above, under, and around itself. Other methods, such as SlackPack^44^, eliminate the need for a frame by using a spot-curing strategy, a method in which resin is locally cured only at the anchors, thus fixing the length and angle between fiber segments. This allows the geometry to be formed as a deployable structure. Its key innovation lies in establishing the spatial relationship between anchors and fibers through a sequence of positioning, tensioning, fixing, and slacking the fibers, in a frameless setup.

**Fig. 1:**
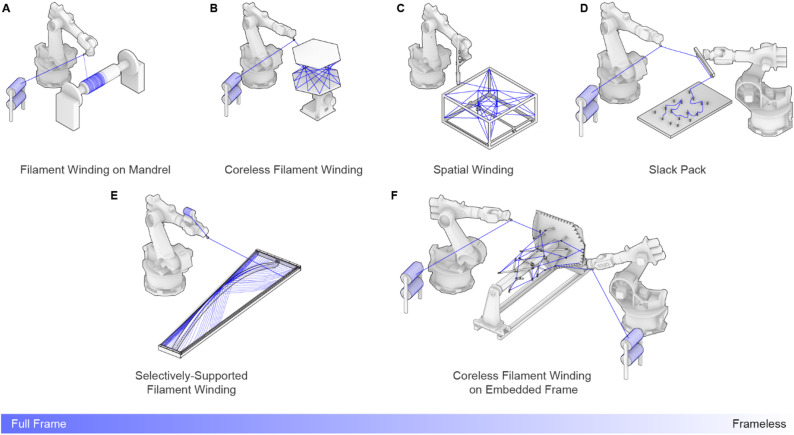
Different filament winding techniques and the level of influence the frame plays in the geometry formation. (**A**) Filament Winding^34^. (**B**) Coreless Filament Winding^35^. (**C**) Spatial Winding^42^. (**D**) Slack Pack^44^. (**E**) Selectively-Supported Filament Winding^28^. (**F**) Coreless Filament Winding on Embedded Frame.

Figure 1 illustrates the evolution of the relationship between frame and geometry in CFW over the years, expanding the design space of fiber structures in architecture and giving rise to various design and fabrication strategies. One approach that remained unexplored is the integration of the frame into the final structure, which could further reduce costs and eliminate waste after project completion. This research investigates this alternative through a new system in which timber elements act as an embedded frame, supporting the fibers during and after the winding process (Fig. 1F).

### Dual-robot fabrication

Previous projects in the AEC domain have highlighted the potential of robotic fabrication processes within the built environment. By transitioning from the use of a single machine to multi-robot systems, these processes can offer various advantages, including enhanced fabrication efficiency, time reduction, and the expansion of the workspace to accommodate the production of larger or more complex building elements.

Wagner et al.^45^ illustrate the versatility and extensive capabilities of a robotic construction platform designed for timber fabrication. This setup features two industrial robotic arms along with an additional positioner. The robotic arms are equipped with custom end-effectors, allowing them to perform a range of tasks such as gripping, nailing, gluing, and milling. Sharing a common workspace, the two machines collaborate to perform the diverse tasks necessary for the production of a timber cassette. Parascho et al.^46^ explored cooperative robotic construction using two industrial robots to assemble a spatial metal structure from discrete steel tubes. Their method involves the alternate positioning of building members, where one robot stabilizes the assembly while the other positions tubes, forming triangulated configurations. Due to complex geometric interdependencies and mutual operational limitations, robotic simulation and path planning were crucial to the design process. Thoma et al.^47^ demonstrated cooperative robotic approaches for timber frame construction. This includes methods for the robotic prefabrication of spatial timber modules in which fabrication and assembly are merged into a unified workflow. The system employs coordinated multi-robot assembly for spatial structures with non-planar geometries, reducing the need for scaffolding while enabling precise, digitally informed construction. Hua et al.^48^ present a cooperative robotic fabrication approach for processing irregular natural wood. In this setup, one robot stabilizes and repositions components while the other performs visual localization and machining.

When viewed together, these examples highlight how multi-robot systems are increasingly leveraged to mediate between geometric complexity, material irregularity, and fabrication precision. In dual-robot coreless filament winding, similar challenges arise: the robots must coordinate motions within a shared workspace, negotiate tensioned fiber paths, and respond to dynamic geometric constraints imposed by the winding logic and the evolving physical fiber net. Collectively, these cases underline a broader trend in cooperative robotic construction: multi-agent systems enable fabrication processes that would be difficult or impossible for a single robot, particularly when dealing with intricate geometries, material variability, or fabrication sequences that require simultaneous stabilization and manipulation.

In the context of CFW, multiple projects have explored the fabrication with more than one machine. The ICD/ITKE Research Pavilion 2013-14^35^ reversed the CFW process by using the winding frame as the robot end-effector, paired with a stationary fiber nozzle. This approach employed two industrial robot arms to minimize the reconfigurable scaffolding and increase the geometric freedom. The robots are fully synchronized in a leader-follower setup in which the follower follows a base plane kinematically linked to the leader. Essentially, the robots execute synchronized motions to maintain the parallel alignment of the two parts of the winding frame throughout the entire winding process. Felbrich et al.^49^ proposed lightweight, long-range machines like unmanned aerial vehicles (UAVs) with precise, strong, but limited-reach industrial robots, achieving a scalable setup for fabricating long-span fiber composite structures. Both projects focused mainly on extending the reach of the robotic setups while placing a single fiber bundle per winding sequence. A simpler heterogeneous multi-robot cooperation was presented by Duque Estrada et al.^50^ for the spatial winding of filament materials. It involves a six-axis robotic arm working in synergy with a customized 2 + 2-axis CNC gantry system, enabling a process of sequentially arranging fibers in space through direct filament interlocking, facilitated by a handover of the fiber bobbin. This approach minimized reliance on robotic reach and formwork but was limited in terms of deposited material amount due to the trade-off between bobbin size and dexterity.

Building upon this research, the proposed dual-robot fabrication methodology leverages the abilities of two cooperating machines to deposit two independent but interacting filament bundles at the same time. This not only increases fabrication efficiency but also extends the design space of possible fiber winding logic beyond what is possible with a single fiber bundle.

This research showcases a hybrid system that combines the complementary properties of timber and flax fibers into a bio-based load-bearing structure. The proposed design and fabrication methods are tailored to enhance their combined strengths, balance material use, and expand architectural possibilities. Timber’s compressive strength and easy workability are strategically paired with the tensile capacity and versatility of natural fibers, enabling new approaches to the design and construction of lightweight hybrid structures. The study aims to build upon the CFW technique by proposing the use of timber as a structure and an embedded winding frame. Additionally, it presents the implementation of a parallel dual-robot winding setup, advancing this approach to an architectural scale. The hybrid system is the result of an in-depth investigation conducted by researchers and students from the ITECH Master’s program, focused on the morphological possibilities arising from the combination of timber and NFPC. The system concept integrates both materials, considering their formal, fabrication, and structural opportunities to form large-scale building elements. The research is demonstrated through a case study centered on the design and construction of a research pavilion, which serves as a testing ground for the proposed methods.

## Methods

This paper presents the computational design methods developed to address the distinct characteristics and constraints of each material within the hybrid system. The following sections outline the approaches to hybrid system logic, form-finding processes, structural analysis strategies, and fiber syntax design, as well as the methods for the dual-robot winding technique. While some methods are described at a general level, others are illustrated through the research pavilion case study, which serves as a concrete example of their application and integration.

### Hybrid material system

#### Material roles

**Fig. 2:**
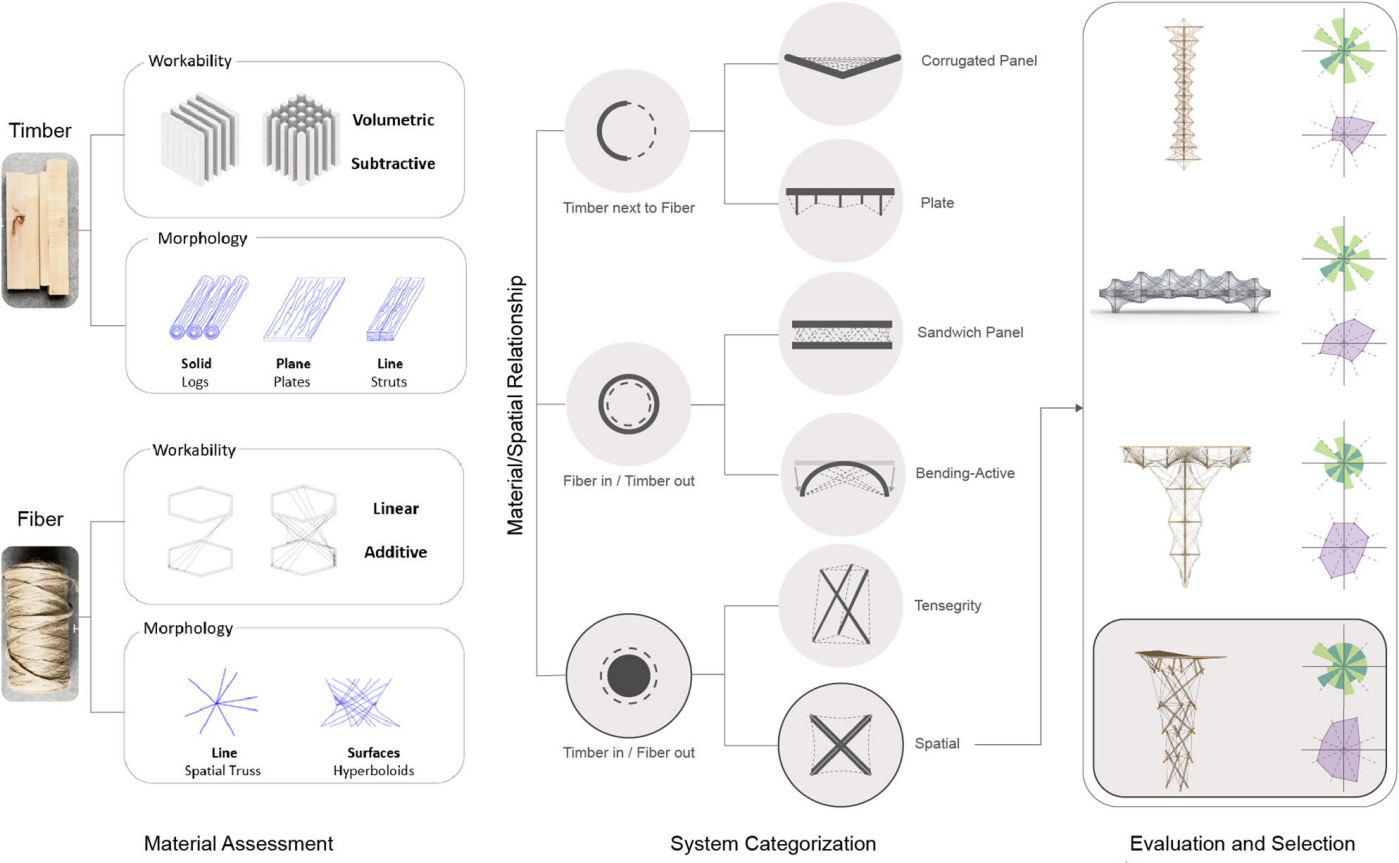
Morphological exploration workflow: material assessment reveals potential complementary properties, followed by an initial physical and digital exploration. From these, key material–spatial relationships are observed, followed by a typology categorization. The emerging system typologies are then refined and evaluated. The selected system is then further developed into a fully architectural system.

In a hybrid system, the material’s roles not only define their main functions but also determine how each material relates to one another spatially. These roles can emerge through two complementary approaches: a top-down strategy, where the material’s strongest properties guide the initial design and structural integration, and a bottom-up process, where new features or functions arise organically during morphological explorations. An iterative feedback loop contemplates architectural, structural, and fabrication opportunities in search of the solution with the highest potential for further development. The compressive strength of timber and the tensile capacity of fibers are strategically exploited to maximize material efficiency while minimizing material consumption. The aim is to develop a system with a high level of interdependency between materials on structural, architectural, and fabrication levels, highlighting their integral contribution to the system as a whole. Another valuable feature strategically explored is the capacity of timber plates to provide shelter, considering the importance of enclosure in architectural projects. This way, external materials can be avoided, and more integrated solutions can be explored. Figure 2 illustrates the initial morphological exploration workflow conducted in the studio, which begins with material assessment, where material properties are collected and examined, followed by the definition of distinct material–spatial relationships. The process then branches into different system typology categories. Each resulting hybrid solution is then further refined and evaluated using the methods presented in^19^.

From a fabrication perspective, this morphological exploration gave rise to another feature. Timber’s easy workability enables the milling of the plate’s edges to become an interface with the fibers. In the traditional CFW process, the fibers are placed around mechanical anchors fixed to a temporary structure, known as a winding frame, typically made of steel. In this case, the timber itself replaces the temporary steel frame, becoming an embedded frame for the fibers. During the fabrication process, the timber supports the fibers, and once they are cured, both materials work together, becoming mutually supportive and essential in maintaining the structure’s spatial integrity. This approach extends the timber’s functionality beyond its traditional structural role, improves off-site fabrication efficiency, and minimizes manufacturing waste.

The morphological exploration led to the development of an expanded co-design framework to guide the design and evaluation of hybrid systems^19^. The evaluation was structured across four domains: form, architecture, structure, and construction; each assessed through domain-specific guiding questions and represented within a quadrant-based diagram, where qualitative judgments were translated into a normalized scoring scale. The framework provided the theoretical background and the tools to support the development of hybrid systems with balanced material responsibilities across different domains. After exploring several design iterations, the main system logic was selected and further developed to enable its full-scale materialization.

### Material choice and characterization

Both planar and linear timber products are considered for material selection, with a combination of softwood and hardwood chosen to optimize structural performance and material efficiency. For planar elements, the focus is primarily on softwood three-layer plates, which are thinner than standard construction CLT and are typically used for ceiling or finishing applications rather than structural purposes. These plates are more cost-effective than construction-grade CLT and are widely available in Germany. For linear elements, hardwood beech is selected due to its high mechanical performance and is utilized when the local structural demand is higher.

The selection of fibers prioritizes flax over other plant-based fibers due to their superior mechanical properties^51^ and local availability^52^. Specifically, Safilin Flax Low Twist Roving TEX 2000, with a twist per meter (tpm) of 20, is chosen for its balance of mechanical performance and availability. According to the product datasheet, the Safilin dry flax roving has a density of 1.44 g/cm^3^, a tensile strength of 212 MPa, and a tensile modulus of 12 GPa. For the composite matrix, a partially bio-based epoxy resin is selected to reduce reliance on petroleum-derived feedstock. Compared to fossil-based epoxy resins, bio-based alternatives incorporate a higher fraction of raw materials from renewable biomass. However, this often results in reduced mechanical performance, including lower strength, stiffness, and glass transition temperature (*Tg*). Sicomin’s Greenpoxy 56 with hardener SD4770 is chosen as the matrix (resin hardener weight ratio 100/30) due to its superior stiffness, *Tg* of 82 °C, and 39.2% bio-based content, as determined in a previous study^28^. However, in that study, Greenpoxy could not be used due to overheating issues after reaching its pot life in a contained impregnation system. To overcome this challenge and facilitate the use of this partially bio-based resin, the fabrication setup proposed in this project employs an open resin bath.

### Hybrid system design

#### Hybrid system logic

The selected hybrid system, resulting from the morphological exploration, unfolds into two component types: columns and roof plates, enabling the structure to expand both vertically and horizontally. The column design draws inspiration from the principles of tensegrity structures, whereby distinct material roles are assigned to compression and tension members according to their respective mechanical strengths. However, in contrast to the relatively low transverse stiffness typically associated with such systems, NFPC possess the capacity to also accommodate mild compressive forces, thereby significantly enhancing the practical applicability of this design approach within a built structure. As a result, the system extends beyond a classical tensegrity model: the fibrous network not only acts in tension but also braces the structure against lateral loads by locally engaging in compression.

**Fig. 3:**
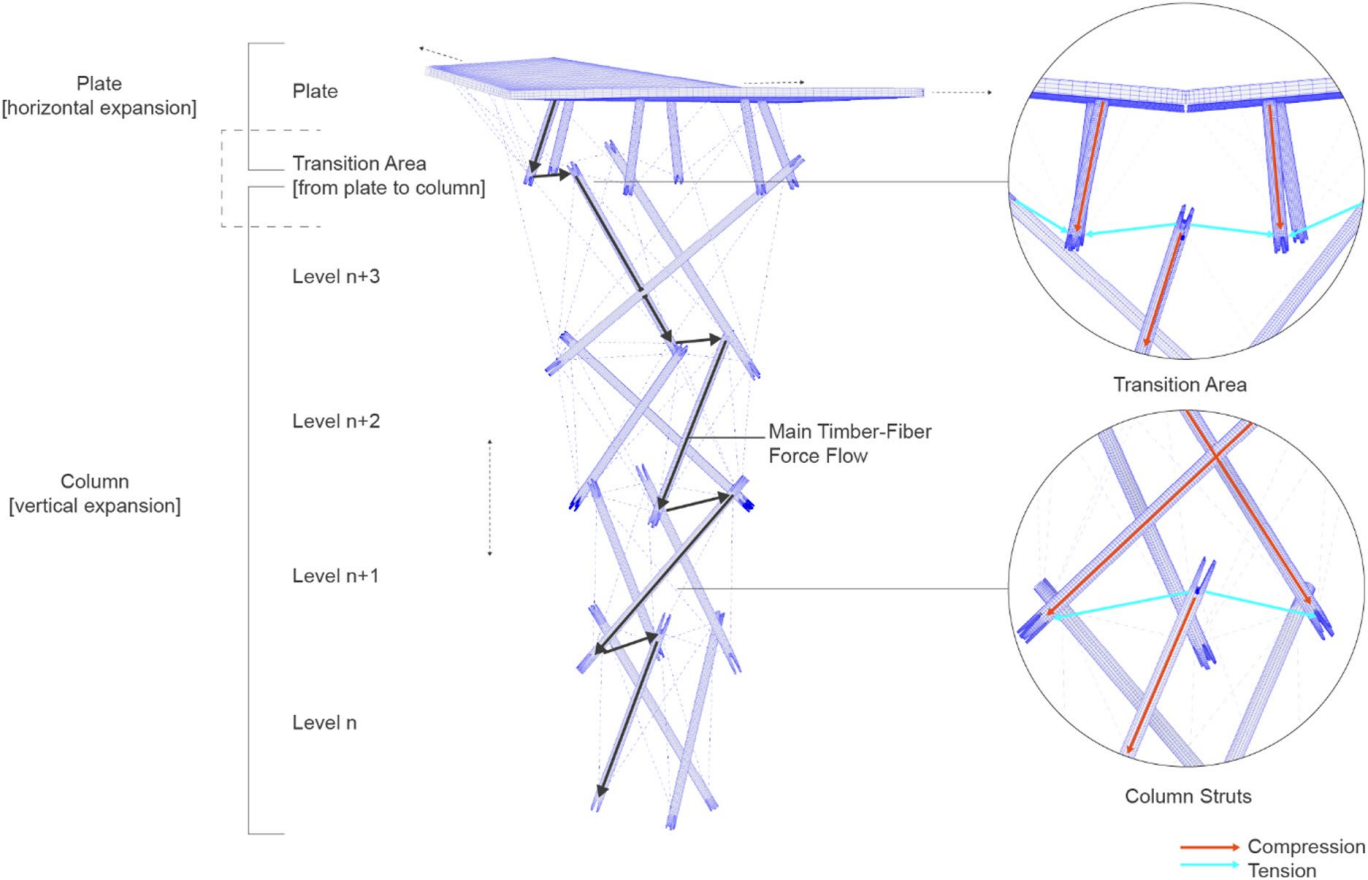
Hybrid system logic. Timber struts are arranged spatially, transferring loads to the NFPC. Timber struts work as compression rods while fibers are mostly under tension.

The columns are formed by placing groups of three timber struts in a radial spatial arrangement along the column height (Fig. 3), in which timber struts function as compression members, while the primary zigzag fiber configuration acts as the main tensile reinforcement, transferring incoming loads as tension to the lower struts. Secondary fiber patterns support this behavior by assisting in the transfer of compressive forces toward the supports and providing lateral bracing for the overall column component. This arrangement leverages the anisotropic properties of both timber and NFPC to guide loads efficiently through the structure. A key aspect of the system is the vertical overlap of successive radial strut levels, which generates the characteristic zigzag geometry, enabling the struts and specific fiber segments to work in compression, while strategic parts of the fiber body are activated in tension under different loading scenarios. The secondary fiber layers further contribute to global structural stability by increasing redundancy and locally decreasing the buckling length of fiber segments.

The roof plates are composed of a set of timber struts fixed under a timber plate. The NFPC forms parallel fiber cords, tensioned by the vertical timber struts, transferring the tensile loads longitudinally across the span. A fiber lattice layer is then added to address the diagonal shear force and brace the components in the short direction. The position, angle, and length of the struts are defined through a form-finding process detailed in the following subsections. The system allows the timber plate and struts to be primarily under compression and the bottom fiber network under tension. The final spatial arrangement between timber and fiber illustrates the distribution of forces in a constant interplay of material interdependence and collaboration.

**Fig. 4:**
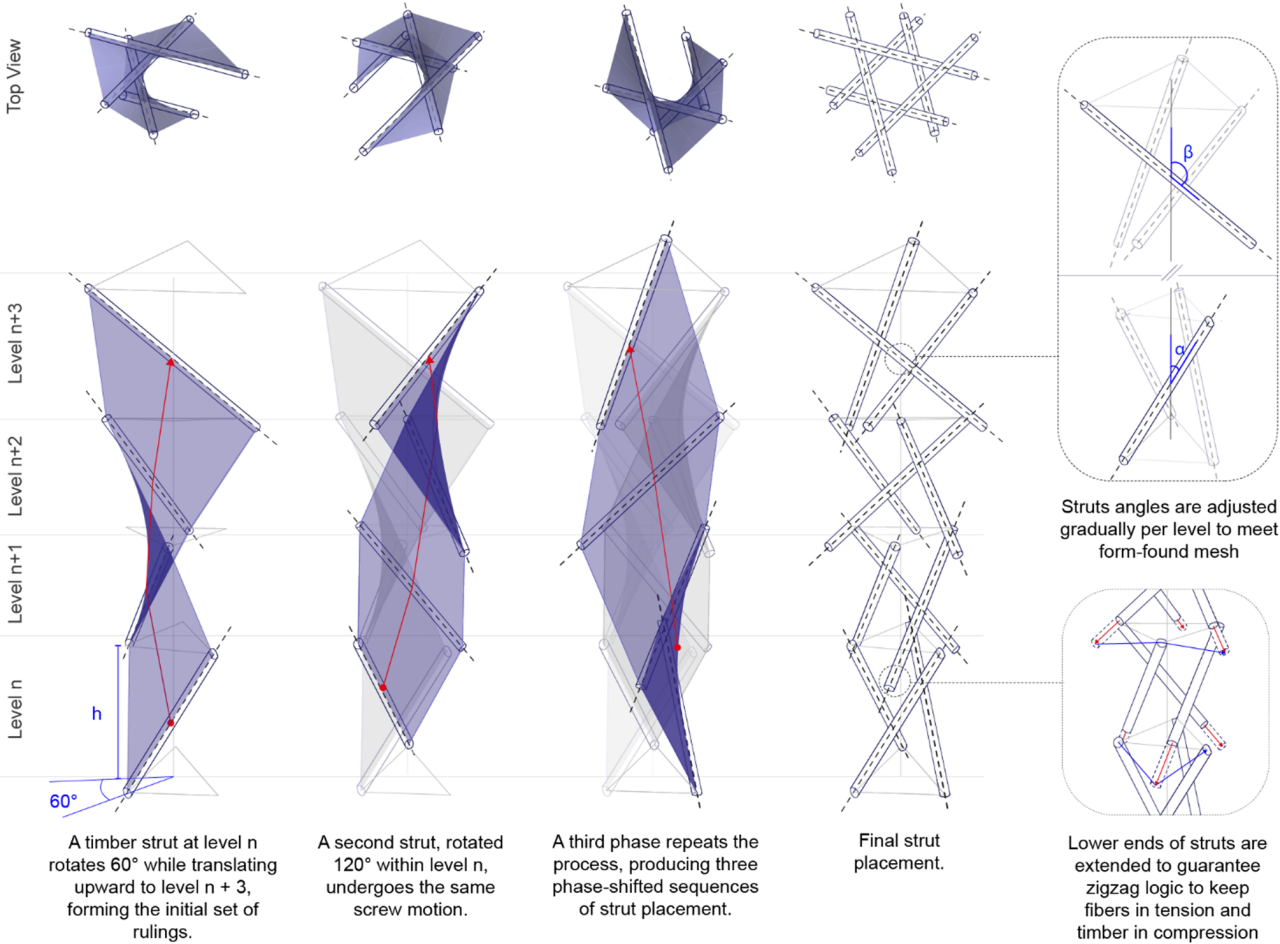
Geometrical logic to form vertical elements. Timber struts are placed radially and undergo a screw motion, repeated several times, until the desired height is reached. Struts angles and lengths are also adjusted to enable the desired transfer of forces.

The arrangement of timber struts in the columns follows a set of rules to guarantee that forces are transmitted as intended and that all elements can adjust to global form-finding (Fig. 4). The configuration is generated through a sequential three-step process based on the repeated application of a screw transformation that combines rotation and translation along the same axis. In the first step, a timber strut positioned at level n and oriented at angle α acts as the initial ruling. This strut rotates 60 degrees while translating upward through the subsequent levels, from *n* + 1 until it reaches the desired height, establishing the primary ruled trajectory. In the second step, a new strut is introduced at the same base level n, rotated 120 degrees in-plane relative to the first, and then subjected to the same combined screw motion, producing a second, phase-shifted set of rulings. The third step repeats this procedure with another strut at level n, creating a third sequence of rulings. Together, these three phase-shifted transformations generate a coherent distribution of struts that collectively approximate the path of ruled surfaces. After all the struts are in place, they undergo some adjustments. The lower ends of the struts extend below the level of the top ends of the previous level, enabling the desired zig-zag configuration. Then the angles between struts and the central axis also change, enabling struts to be perpendicular to the form-found fiber mesh.

#### Design-to-assembly workflow and data exchange

**Fig. 5:**
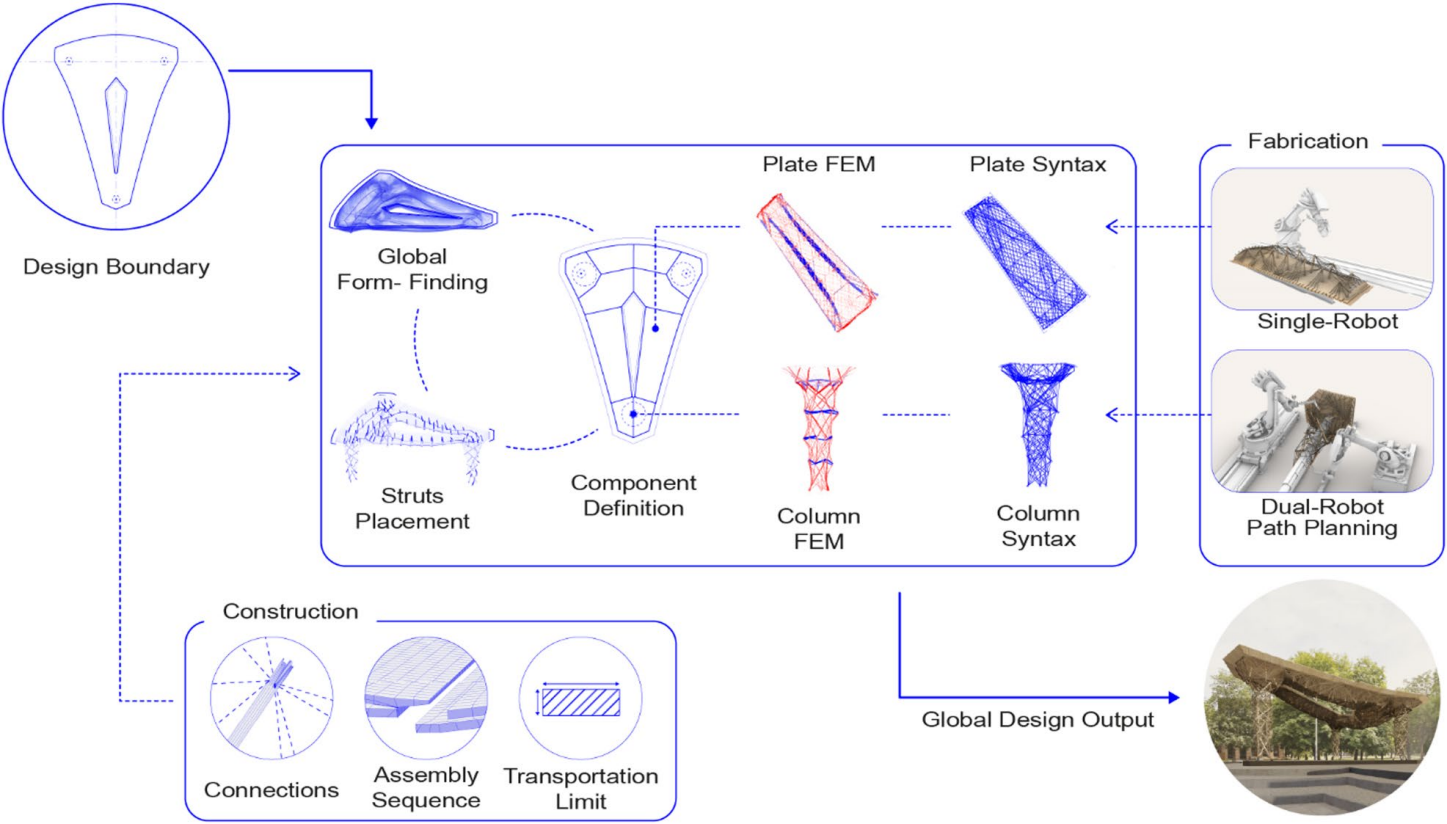
Design-to-assembly workflow.

The proposed system requires constant negotiation between fabrication constraints, material characterization, and structural performance. In order to streamline the design process and enable the interoperability of reciprocal data between different domains, an integrated co-design approach was implemented, synthesizing the developed concepts into an iterative digital workflow. The workflow encompasses inputs and outputs from global geometry, structural analysis, syntax design, joints, and fabrication (Fig. 5).

The design workflow integrates global design boundaries, component segmentation, syntax design, structural assessment, and fabrication, all within a multidisciplinary co-design framework^29^. Initially, design boundaries are defined by considering spans, available material dimensions, and design intentions, which inform the form-finding process and guide the structural depth of the roof plates. This process also establishes the strut placement, angle, and length, and defines the primary fiber directions, influencing the syntax design. Detailed modeling follows, incorporating timber plates, struts, and fiber layup, allowing for a more comprehensive structural analysis through finite element modeling (FEM). The FEM evaluates the components based on material properties and design loads, helping determine the final material quantities required for fabrication, such as timber element thickness and fiber rovings count with appropriate winding passes. Throughout this process, continuous feedback from structural simulations, fabrication constraints, physical tests, and assembly logic informs the refinement of the design, ensuring alignment with both structural performance and fabrication requirements.

Ultimately, the digital workflow generates fabrication files from a streamlined design-to-assembly process, leveraging the individual materials’ potential to create a complex, high-performance morphology. The design-to-assembly workflow happened within the parametric framework provided by Rhinoceros^®^^53^ and Grasshopper 3D^54^ and utilized the distributed Common Data Environment (CDE) Speckle^55^, which provides an open-source version control system and enables the highly collaborative co-design workflow. The methods used within the workflow are presented in the following sections.

#### Form-finding

**Fig. 6:**
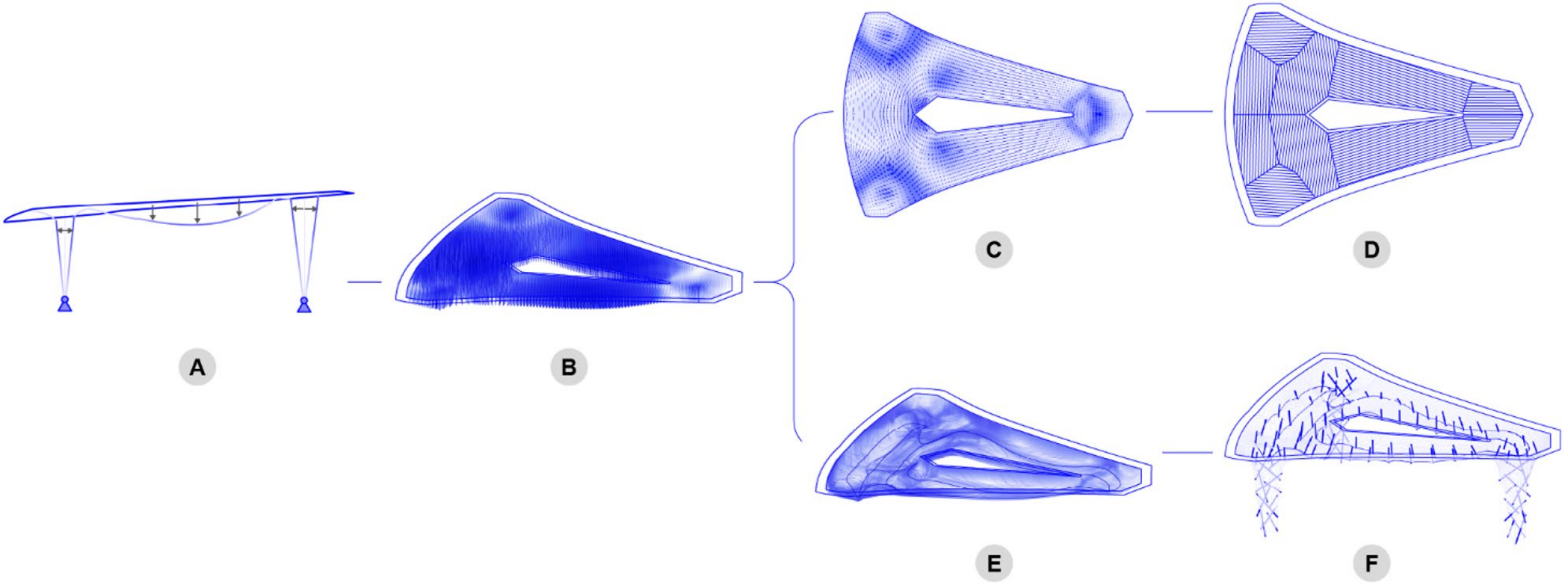
Form-finding process of the hybrid pavilion. (**A**) Global moment magnitudes with simplified surface representation. (**B**) Generation of a fiber mesh with target depth determined by moment magnitudes. (**C**) Extraction of principal stress field from the top surface. (**D**) Timber plate’s grain orientation is informed by the stress field and the components discretization. (**E**) Principal stress field from the fiber mesh informs fiber pattern design. (**F**) Minimizing bending moment in timber struts via local orientation adjustment.

For defined global dimensions, such as the overall structural height, maximum spanning distance, and aperture size, the structural form-finding entails three steps, as illustrated in Fig. 6. A simple roof geometry is modeled as a surface with uniform thickness, analyzed under self-weight with pin supports assumed at the column positions (Fig. 6A). The resulting absolute values of principal moments are used to scale structural depth and define an initial fiber body, represented as a mesh (Fig. 6B). This fiber mesh, combined with a top timber surface, is simulated once again to trace the paths following principal moment directions both on the timber surface (Fig. 6C) and on the fiber mesh (Fig. 6E). The principal moment field on the timber surface informs the plate orientation and segmentation (Fig. 6D), whereas that on the fiber mesh informs the fiber pattern design. Timber struts are distributed along the populated paths, oriented perpendicularly to the fiber mesh, and scaled in accordance with the target structural depth. The final shaping of the fiber body is then approximated by dynamic relaxation, and the columns are formed at the support locations following the system logic presented. Finally, a third simulation loop is carried out to iteratively reduce the local bending in the timber struts via fine-tuning of the individual strut orientations (Fig. 6F), resulting in a final geometry enabling efficient stiffening of the hybrid structure.

#### Syntax development

Derived from global design and structural form-finding, components can be categorized into two main types: vertical supporting columns and horizontal roof plates. The syntax, a notational system that describes the winding pattern through a list of sequential anchor points^56^, is developed in accordance with the geometry and structural functions of each component. Through the syntax, different layers will form fiber layups, bearing the structural loads within and between components.

**Fig. 7:**
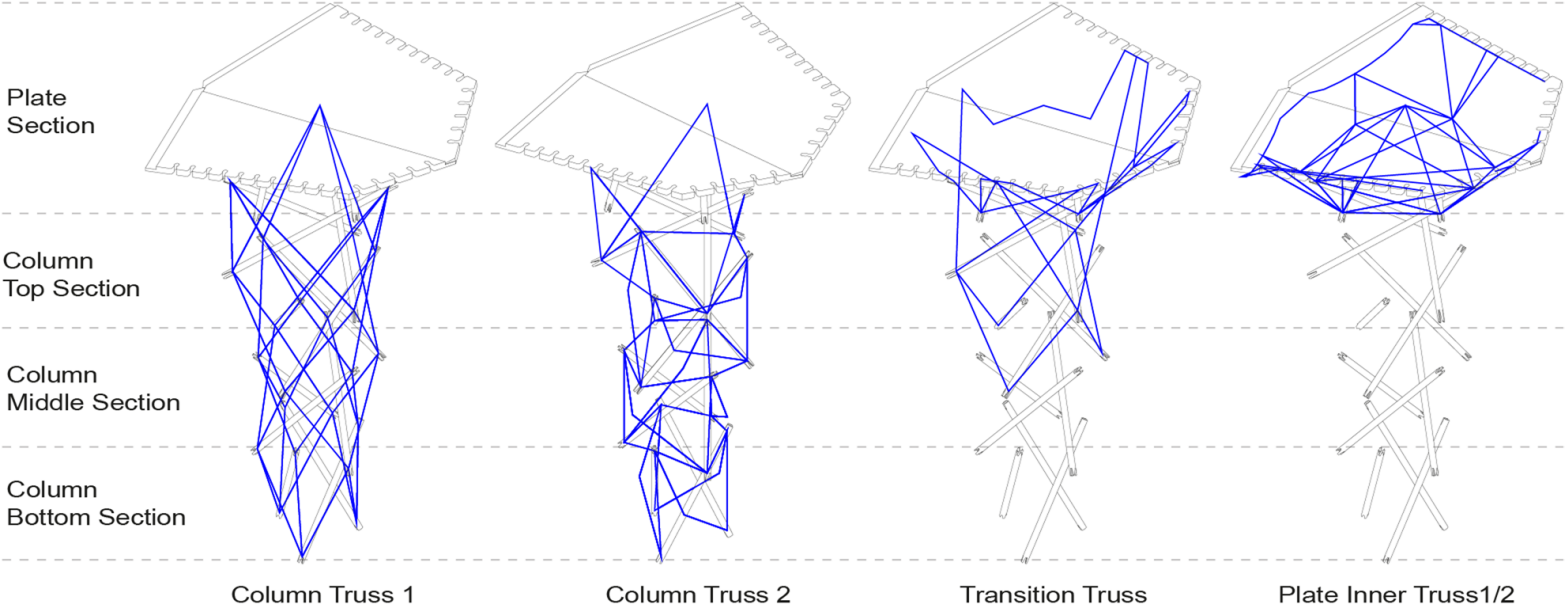
Main structural layers of the column syntax.

The columns’ layup incorporates four main fiber layers as shown in Fig. 7. A vertical fiber truss (*Column Truss 1*) runs along the column height, connecting the struts and transferring compression forces downward in conjunction with the timber elements. A second fiber truss forms a zigzag pattern, providing the horizontal ring with fibers in tension, bracing the vertical fibers as lateral restraints (*Column Truss 2*). To facilitate the connection between horizontal plates and vertical columns, a transition truss (*Transition Truss*) extends from the top plate edge into the column’s middle section, balancing lateral loads from the plates. Finally, a horizontal inner truss (*Plate Inner Truss 1/2*) stabilizes the expansion of the column head section, enhancing structural integrity.

**Fig. 8:**
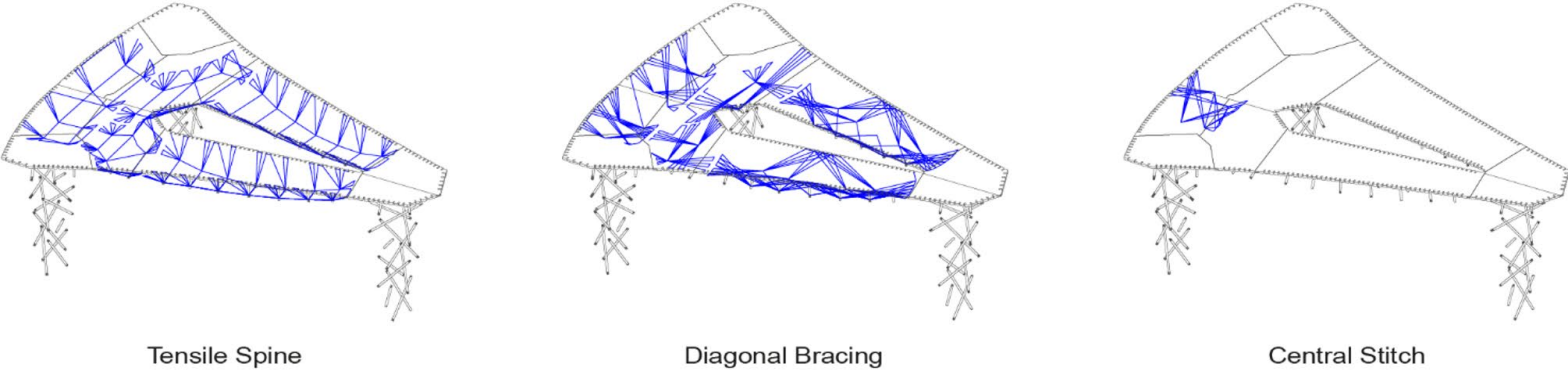
Main structural layers of the plate syntax.

The plate syntax addresses two primary loading conditions within the fiber body and two types of component connections (Fig. 8). Based on the form-finding results, the suspended plates are categorized into one-way plates crossing the long spans and two-way plates linking these spans. Areas of higher bending, particularly at the midpoints of long spans, require a concentrated tensile layer (Fig. 8, left). A diagonal bracing layer (Fig. 8, center) enhances stability, strengthens the fiber body, and distributes forces at the plate edges to minimize stress on timber and fiber connectors. The two-way plates follow the same logic, incorporating a tensile spine and bracing layer for load distribution. Additionally, the long-span plate between the back columns is composed of two symmetric angled sub-plates, reinforced by a central stitch syntax (Fig. 8, right) to strengthen the connection under high bending forces.

**Fig. 9:**
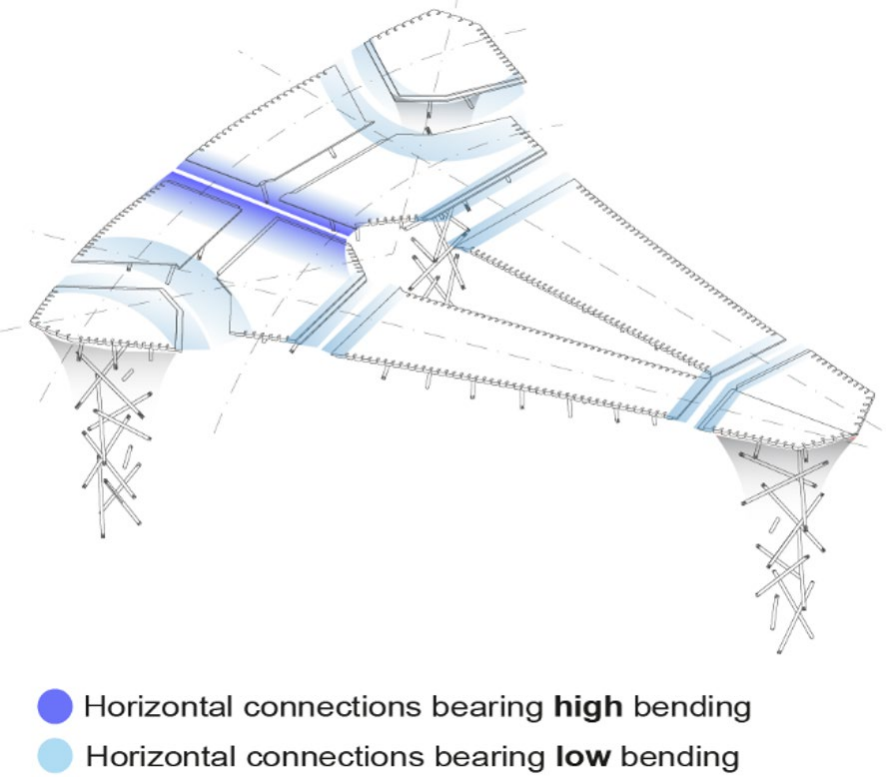
Connection logic following bending stress distribution.

Figure 9 illustrates the distribution of bending stress between components. In plates, most of the horizontal connections marked by light blue deal with low bending moments, while a special connection marked in dark blue needs to be achieved by the syntax under tension due to the high bending in the long-span plate. These high and low bending areas will inform the design and dimensioning of connections.

### Material interfaces

**Fig. 10:**
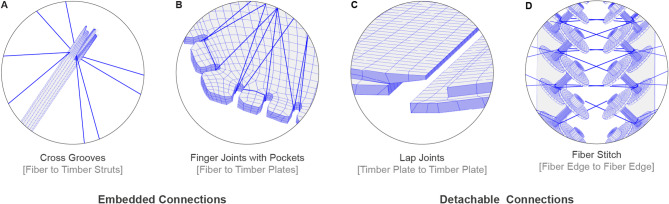
Detail of connection types. (**A**) Cross grooves connecting fibers to timber struts. (**B**) Finger joints connecting fibers with timber plates. (**C**) Lap joints connecting timber plates between components. (**D**) Fiber stitch connecting fiber edges.

A defining characteristic of hybrid systems is the intrinsic prerequisite to bring different materials together, and the way these connections are established directly influences the fabrication process. To integrate the hybrid system, two levels of connections are required: one between materials (fiber-timber) and another between components (timber-timber and fiber-fiber). A total of four different joints are developed, as seen in Fig. 10.

Following the strategy of using timber as a frame, two fiber-timber connections are developed, one between fibers and struts and the other between fibers and plates. Both are classified as embedded connections, meaning they are integrated during fabrication and cannot be easily separated. Given the machinability of timber, the struts are milled with a deep cross groove (Fig. 14A), allowing fibers to pass through from four directions (Fig. 10A). These grooves serve as anchor points, securing the fibers during the winding process and after curing. The depth of the groove is critical: if too shallow, fibers may accumulate outside the groove, compromising positional accuracy during winding; if too deep, it can weaken the timber and may lead to undesirable aesthetic outcomes.

**Fig. 11:**
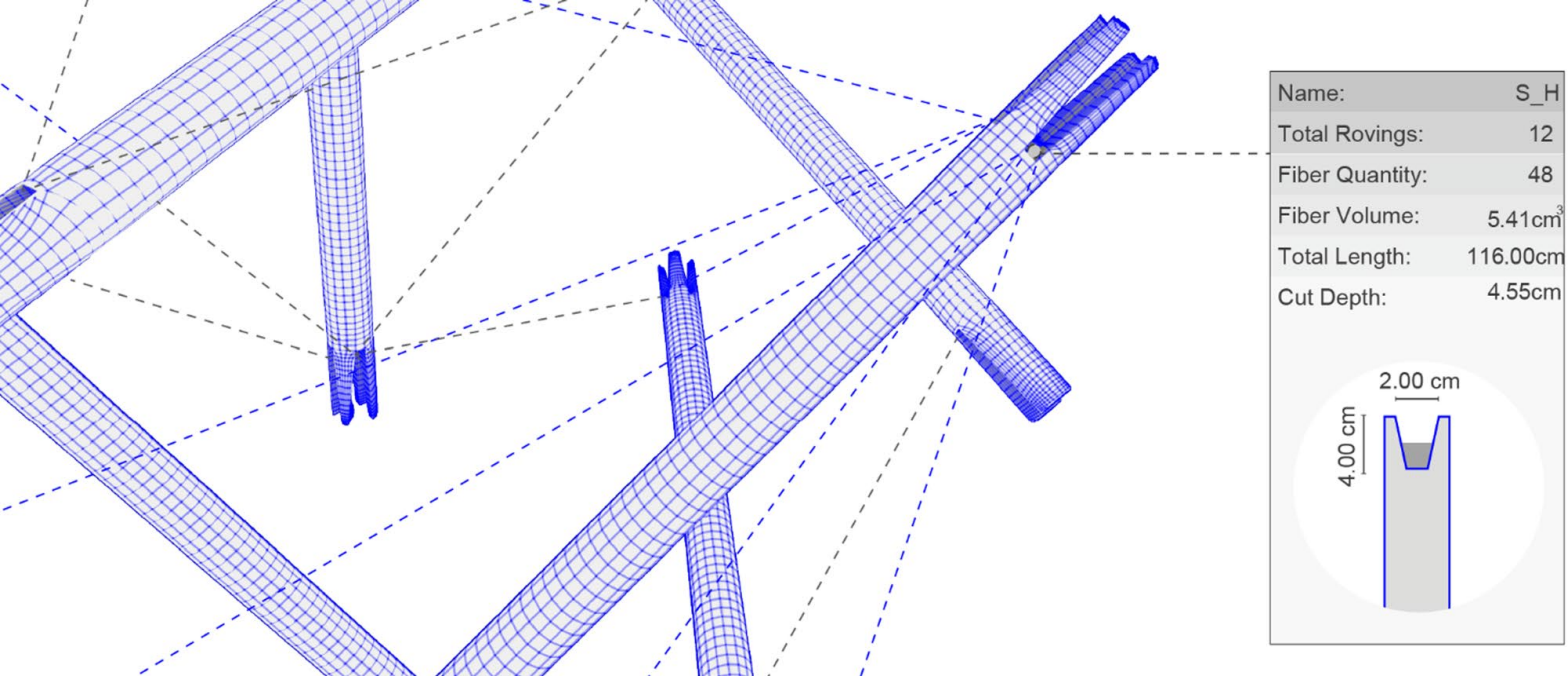
Timber struts metadata: Fiber volume for each strut end-cap is calculated, informing groove depth.

To address this, a method is developed to calculate the groove depth based on the minimally required fiber volume. Within the digital modeling environment, all anchor points located at the strut ends are assigned an ID and corresponding metadata that records the number of fiber segments passing through (Fig. 11). Using this information, along with the total fiber rovings in the bundle and the rovings density, the fiber volume within each groove is calculated, determining the appropriate milling depth and the final length of each strut.

The second fiber-timber connection occurs between the fibers and timber plates. In this case, finger joints are customized by incorporating a pocket to accommodate the fibers during and after the winding process (Fig. 10B). This modification allows the finger joints to work as normal timber-timber joints while providing a secure interface for the fibers. To achieve a visually consistent result across the milled plates, an average fiber volume passing through each pocket is calculated, and a uniform pocket size is applied to all joints.

At the component level, two reversible connection systems are introduced to enable efficient on-site assembly and disassembly. The first system uses lap joints, which connect timber plates through cross screws, ensuring structural stability (Fig. 10C). The second system, called fiber stitches, incorporates a fiber-fiber connection, designed to facilitate a direct force flow between fiber composite elements at the interface of connected components (Fig. 10D). The dimensions of the lap joints are determined based on the minimum edge distance required for the selected screw diameter. To optimize the screw placement and spacing, force values required for load transfer between timber plates are obtained from the global FE simulation. These values inform the selection of screw diameters and the maximum allowable spacing between pairs of cross screws, ensuring effective load distribution while maintaining the reversibility of the connections.

Fiber stitches are developed to connect the lower part of the component edges ending in fibers (Fig. 14B). To enable this, temporary timber frames, designed to match the predefined structural depth, are fixed onto the plates. Steel bolts, secured with threaded inserts, are positioned along the frame edges to accommodate conventional mechanical anchor points used in CFW. Each anchor point consists of a set of two washers and a steel spacer assembled in a sandwich-like configuration, creating a space for fibers to be wound around. After curing, the temporary frame, washers, and bolts are removed, while the spacers remain embedded as permanent connection points. This exposes the fiber edges, leaving them free for assembly. A novel fiber stitch method is introduced to join these edges. The stitches are pre-wound before final assembly by aligning the completed components and manually winding a pattern across the adjoining edges. The stitches are left to cure until rigid at room temperature and are then subjected to a full curing process in an oven. Once cured, the fiber stitches function as structural connectors, enabling the fiber bodies to be assembled on-site with mechanical fasteners.

### Dual robot winding

The use of timber as a frame requires careful consideration, significantly influencing the coreless winding strategy. Due to the slenderness of the struts, even minor load exertion at one end could cause breakage. Typically, CFW relies on industrial robotic arms to wind fibers around a robust steel frame. The tension applied by the robot varies depending on each setup, but it will rely mostly on the robot’s speed and the resistance of the fibers being pulled. To ensure balanced tension distribution on the struts, a parallel dual robotic winding setup is proposed, enabling a fiber syntax that would not be possible with only a single robot. In this system, both robots work in sync, reaching opposite sides of the same strut to apply even tension on both ends. This strategy is primarily used for the initial layers until all struts are wound on both sides and secured evenly by the fibers. This project represents the first use of a parallel coreless winding setup to fabricate a full-scale structure.

This approach requires the development of a synchronous path planning algorithm, precise calibration of both robots within the same Cartesian space, and the creation of a parallel fiber impregnation system to feed both robots simultaneously. The methods covering these developments are presented in the following subsections.

#### Fabrication setup

**Fig. 12:**
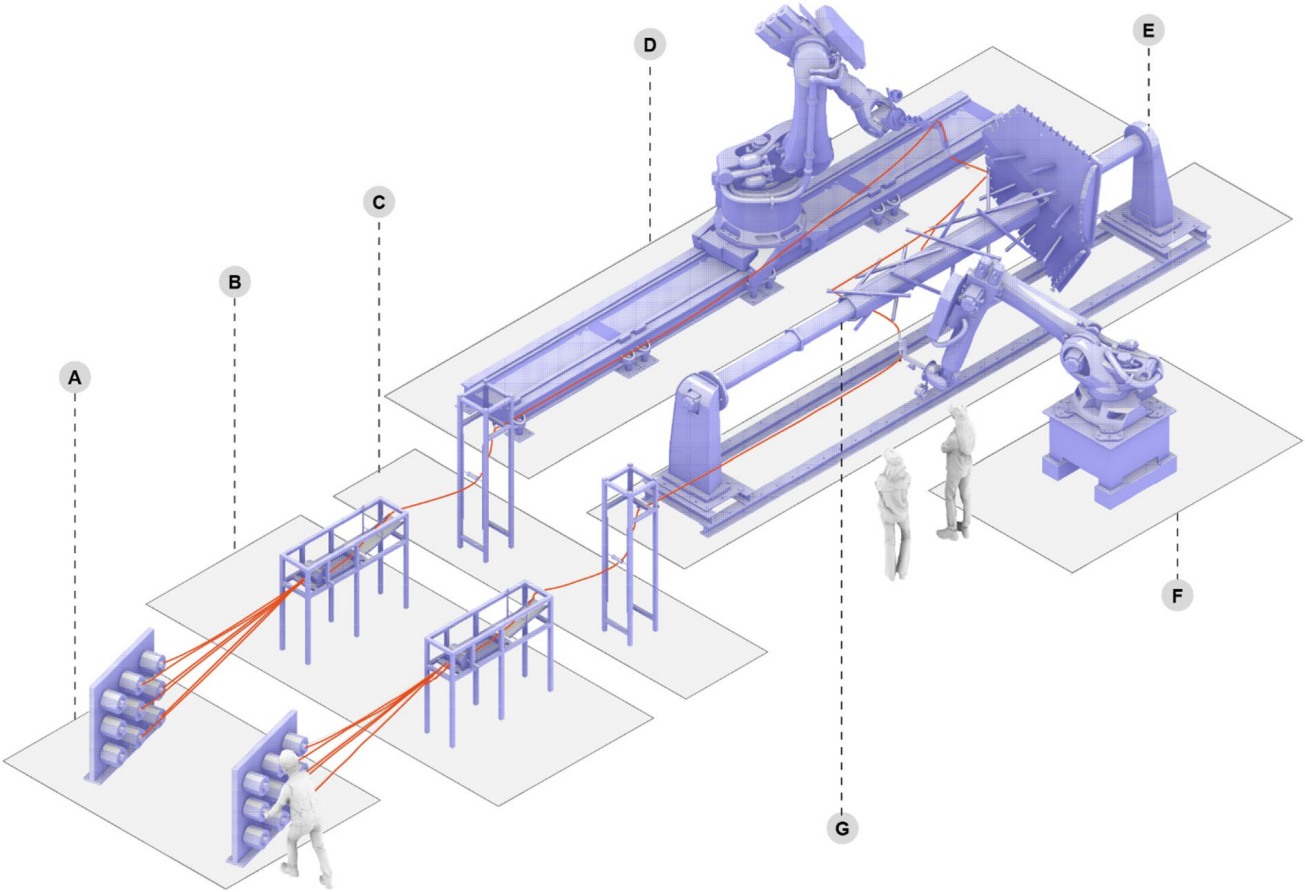
Fabrication Setup. Dual-robot winding with a parallel fiber roving feeding system. (**A**) Spool holder magazines. (**B**) Resin baths for impregnation. (**C**) Tensioning system. (**D**) Robotic arm on a linear track. (**E**) One-axis positioner. (**F**) Robotic arm on a pedestal. (**G**) Embedded frame supported by a steel tube.

For winding the columns, the collaborative setup involves two six-axis industrial robotic arms: one mounted on a linear track and another fixed on a stationary pedestal (Fig. 12). The motions of both robots are synchronized with an external axis, enabling dual winding to occur simultaneously, much like a choreographed performance. The external one-axis positioner, which supports the column during winding, has an active side coupled to a passive one through a steel tube. Two resin baths impregnate the fibers with the matrix, while two spool holder magazines supply the dry fiber rovings to the resin baths.

**Fig. 13:**
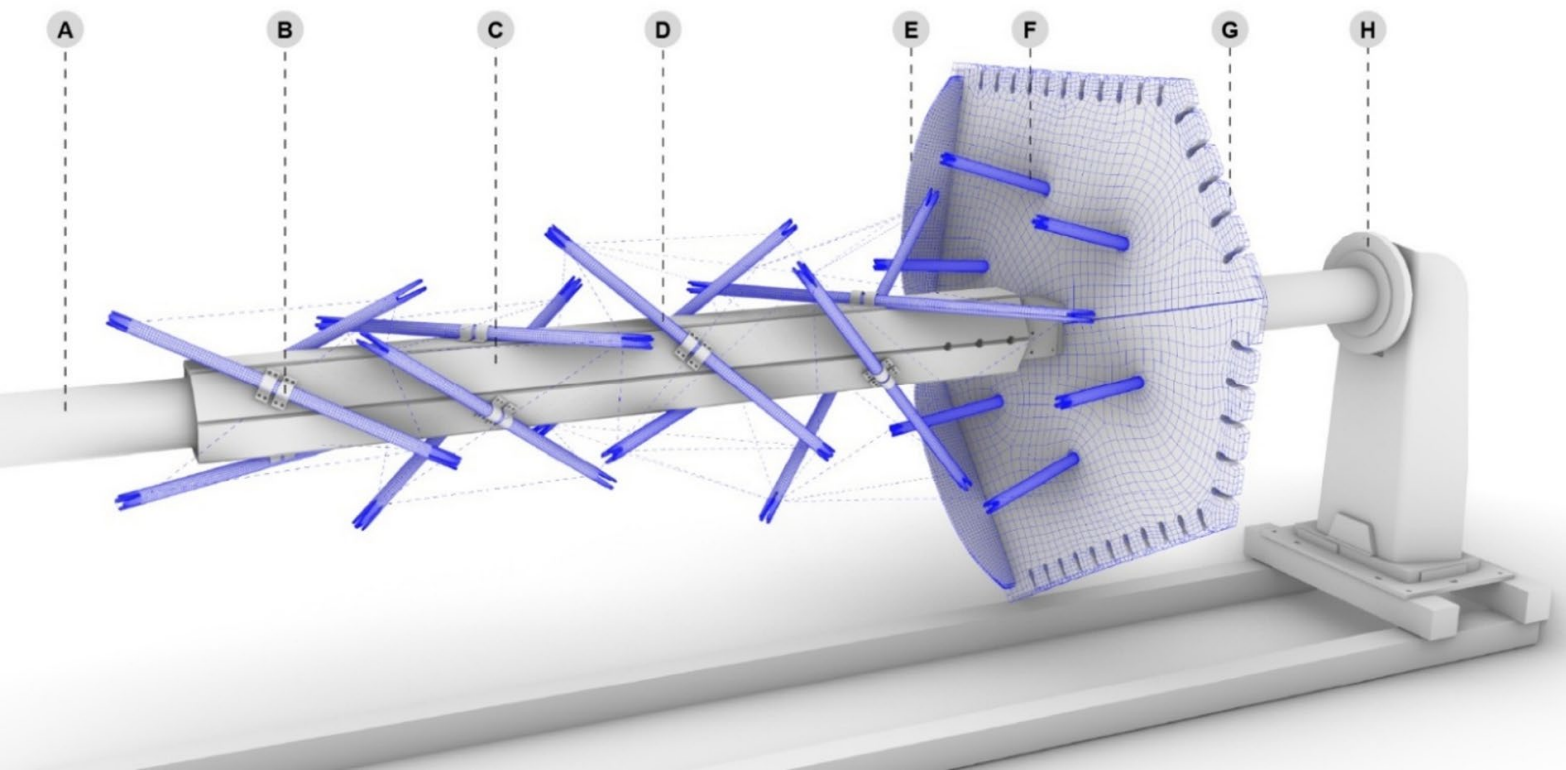
Detail of the column’s embedded timber frame temporarily supported by a steel and timber core connecting the one-axis positioner during fabrication. (**A**) Steel tube connecting one-axis positioners. (**B**) Steel brackets support the timber struts and allow for easy removal. (**C**) Wooden planks containing CNC-milled fixing points for steel brackets. (**D**) Timber struts. (**E**) Temporary frame populated with anchor points. (**F**) Plate struts. (**G**) Timber plate (**H**) One-axis positioner.

To temporarily support the column’s embedded timber frame on the external axis, a set of wooden hexagons is fixed around the steel tube, allowing six long wooden planks to be screwed onto them (Fig. 13). These planks are equipped with threaded inserts, positioned according to the placement of all struts for the three columns. For easy installation and removal of the struts after winding and curing, simple off-the-shelf steel brackets are used to fix them in position on the wooden planks. These brackets fit around the circular cross-section of the struts and feature four fixing points. Given the relatively low density of the final fiber layup, the screws fixing the brackets can be accessed with an extended drill bit for removal. The angles of each strut in relation to the planks are ensured by the CNC-milled bracket fixing points.

**Fig. 14:**
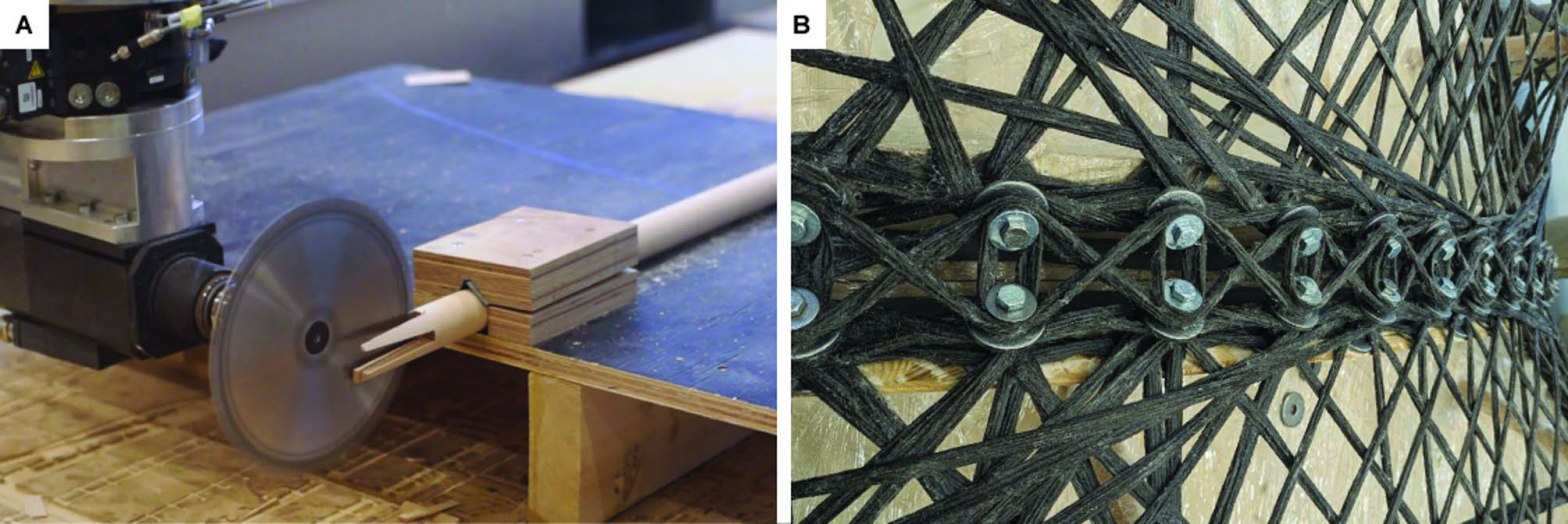
Fabrication details: (**A**) robotic cutting of strut grooves with a saw blade attached to the milling spindle; (**B**) in-place winding of fiber stitches as fiber-fiber connection elements. © ITECH/ICD/ITKE University of Stuttgart.

For the struts fixed on the column plates, their angles are determined by the metadata provided in the digital workflow. The strut grooves are robotically cut to their defined depths with a saw blade attached to the milling spindle (Fig. 14A). Once the cross grooves are milled, the other ends of the struts are also cut at an angle. On the opposite side, the three-layer plates are robotically milled into shape, incorporating finger joints, lap joints, and shallow grooves to accommodate the angled struts. These struts are then fixed in place with wood glue and screws.

A simpler winding setup is used for the hybrid plates. Since one end of each strut is fixed to the plate, it does not require two robots to maintain even tension. In this case, considering the required reach to wind long plate elements, the robotic arm installed on the linear track is used. The struts are fixed to the column plates, with the angled cut fitting into a shallow groove and secured with wood glue and screws.

Both plates and plates on columns received temporary frames on the edges, sharing a component-component connection. These temporary frames are fixed on the timber plates with cross-screws and are populated with a series of anchor points that are wound on, hosting the fibers until cured. The anchor points’ orientation is crucial for maintaining structural integrity during load transfer^57^ and maintaining fibers in place during the winding process^28^. The temporary frames are milled to conform to the components’ cross-section geometry established by the form-found structural depth. The top edges hosting the anchors receive a 45**°** angle cut to improve the stability of the fibers during winding, preventing the fibers from slipping past the washers and providing an accessible angle for the winding of the fiber stitch connections (Fig. 14B).

#### Calibration

To check the manufacturability of the planned building components, a reachability check is performed to evaluate the capability of the robotic setup^30^ and to ensure suitable placement of the second robot and external axis. The shared workspace requires careful consideration of the cell configuration. For the fabrication of the column components, a one-axis positioner is coupled to the robot on the linear track. This positioner is crucial to ensure that all winding planes are reachable by the two robots. It is essential to calibrate the external axis and the robot coordinate systems to provide accurate spatial references for path planning. The positioner’s axis of rotation must be precisely surveyed to align the digital path planning model with the physical setup and workpiece. This alignment ensures that the path planning produces trajectories that correspond accurately to the physical position of the setup. After placement of the workpiece, the winding points of the component are surveyed, and the digital model is updated to reflect the actual positions of the winding points on the physical setup. The path planning automatically generates an updated toolpath.

#### Path planning and synchronization

To define the robot’s trajectory, the fiber syntax must be translated into a robotic toolpath. This translation involves calculating the pose, comprising Cartesian coordinates and orientation, of the tool center point (TCP) on the robot’s end effector as a discrete representation of the desired toolpath. During this process, it is essential to account for the dimensions, positions, and orientations of the anchors, as well as the robot’s workspace and kinematic boundary conditions.

**Fig. 15:**
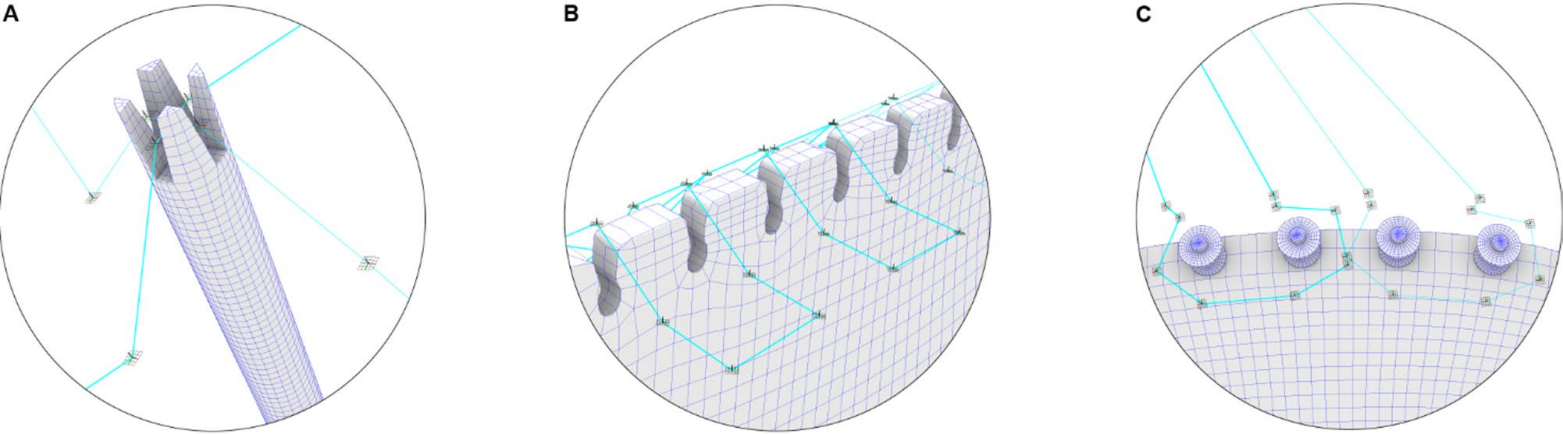
Path planning for robotic winding onto the timber frames: fiber hooking motions for (**A**) cross grooves of the embedded timber struts, (**B**) embedded finger joints, and (**C**) winding anchors with bolts and washers on the temporary frame.

The path planning process utilizes a parameter-based method, as described by Hügle et al.^58^, to generate a sequential TCP path. This generated winding path is segmented into two types of motions: hooking and travel. Hooking motions specify how fibers are looped around anchor points, while travel segments encompass the remaining path segments between these hooking motions. To account for the different material interfaces between fiber and timber (Fig. 10), multiple hooking motions have been implemented (Fig. 15) to wind onto the (A) cross grooves of the timber struts, (B) finger joints, and (C) winding anchors with bolts and washers. Due to the taper in the cross grooves of the timber struts (from 20 mm to 10 mm), as well as the tapered opening and the filleted edges of the finger joints (from 50 mm to 20 mm), these anchor points are able to compensate for the varying incoming fiber angles. By allowing the fiber bundles to slide into position, this approach effectively mitigates any potential tolerance issues during the winding process. The robotic code, written in KUKA Robot Language (KRL), is generated directly from the custom robotic simulation tool.

The syntaxes for the first layers are divided into two distinct winding paths, allocated to the two robots for parallel execution. This requires defining motion synchronization commands to specify winding planes where both robots must arrive simultaneously, ensuring simultaneous execution of the robot paths and preventing collisions or interference between them. To control the cooperation of the two robots and the external kinematic system, the KUKA.RoboTeam package^59^is used. It allows the synchronization of the timing of the robot and program interpreters, as well as their motions.

## Results

The presented methods are demonstrated through a case study that encompasses the design, fabrication, and assembly of a research pavilion on an architectural scale. The results of the methods for design-to-construction are presented in the following subsections.

### Pavilion global design

**Fig. 16:**
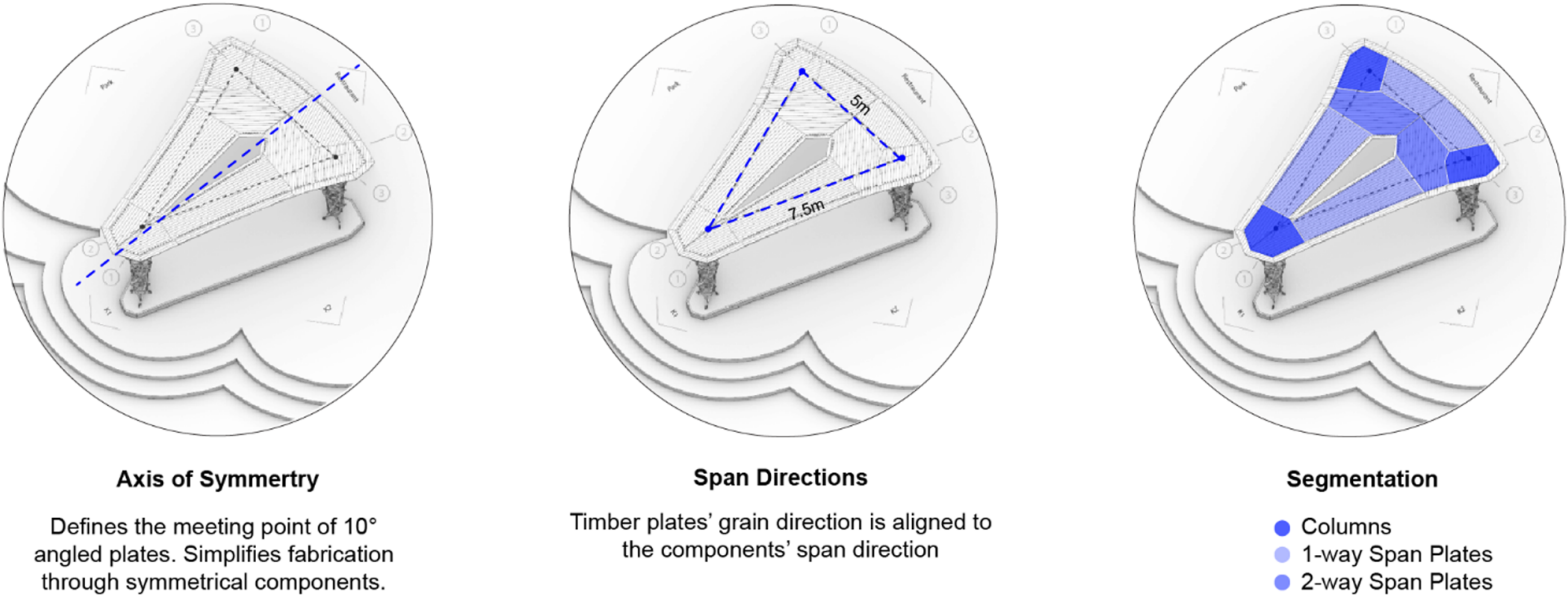
Pavilion global design strategies.

The final pavilion design embodies the interplay between material, geometry, structure, and fabrication, synthesizing the developed methods presented in this paper (Fig. 16). It features a canopy composed of NFPC-timber hybrid elements, including three columns and five plates. This lightweight structure accommodates three single-way spanning and two two-way spanning plates through a highly dematerialized structure. The geometry is discretized to align with timber’s optimal grain directions, target spans, and the maximum available three-layer plate sizes. These components were designed to meet fabrication constraints and converge at a maximum angle of ten degrees to facilitate rainwater drainage.

**Fig. 17:**
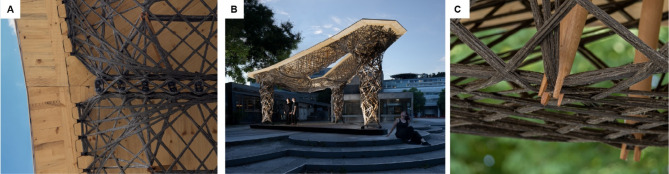
Details and nocturnal view of the finalized research pavilion. @ ITECH/ICD/ITKE University of Stuttgart.

The pavilion is located in the University’s campus park, a place traditionally known for hosting previous research pavilions (Fig. 17). The pavilion sits on a temporary foundation composed of steel beams, concrete blocks, and gravel. The steel ground beams connect the three columns’ bases. The timber struts that reach the bases from the columns are pin-connected to the foundation structure. Concrete blocks and gravel provide the necessary weight to prevent up-lifting forces from wind or live loads. During disassembly, the foundation’s materials are easily separated and reused.

The NFPC-timber hybrid structure exemplifies a high degree of dematerialization and interdependency. Timber and fiber rely on each other for stability and load transfer. This interdependency extends beyond design into fabrication, where timber serves as an embedded frame essential for fiber placement (Fig. 17A–C). Through their complementary roles, the three-layer plates remain slender despite spanning 7.5 m. All roof plates utilize 42 mm, three-layer timber boards, while the timber struts, made from 42 mm diameter beech wood, provide sufficient stiffness to withstand tension during winding and accommodate cross-grooved end caps for secure connections.

### Final syntax layup

**Fig. 18:**
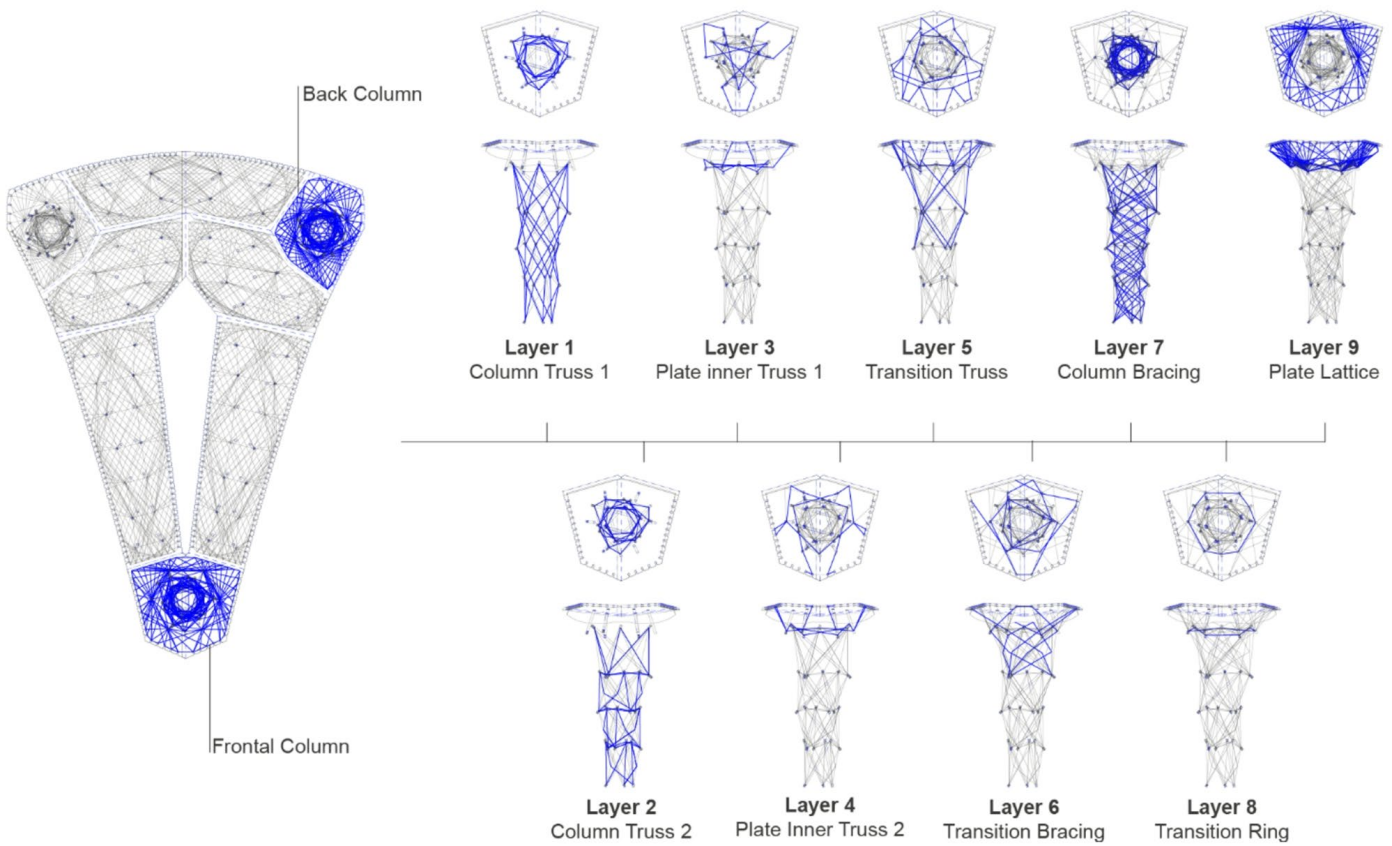
Final fiber layup for frontal and back columns.

Figure 18 summarizes the final syntax design for the columns. All three columns follow the same syntax logic, varying only in rovings and passes between the frontal and back columns. The main structural layers and additional bracing and lattice layers follow the winding sequences from left to right. The number of rovings (R) defines how many fibers are used to form a fiber bundle, while the number of passes (P) defines how many times that layer will be wound (Table 1). Both values are informed by structural analysis and comply with fabrication capabilities. A total fiber roving length of about 6060 m is used for the frontal column, and around 6710 m for each of the two back columns.

**Table 1:** Number of rovings per pass and total passes for final columns layup.

Layer:		1	2	3	4	5	6	7	8	9
Frontal column	Rovings	12	12	10	10	10	10	12	10	6
	Passes	2	1	1	1	3	3	1	1	1
Back columns	Rovings	12	12	12	12	12	12	12	8	8
	Passes	3	3	2	2	3	2	2	1	1

**Fig. 19:**
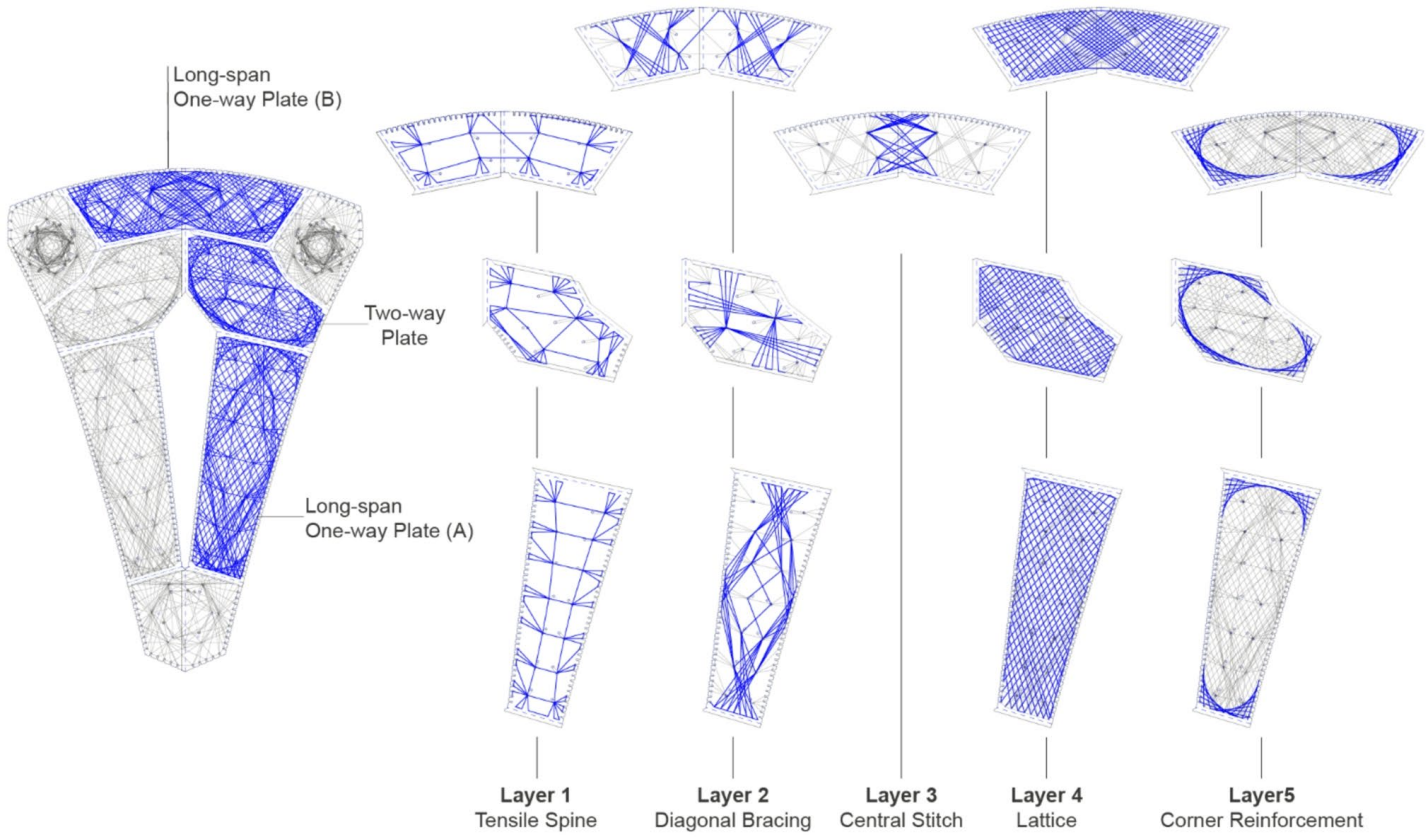
Final fiber layup for one-way and two-way span plates.

Figure 19 shows the final design of the plate syntax following the winding sequences, and Table 2 clarifies the corresponding amount of rovings per pass and pass numbers. The total fiber roving length of the two plates spanning between the front and back columns is about 7760 m. The total roving length of the two two-way span plates is about 4300 m. For the long-span plate between the back columns, the total length is about 3830 m.

**Table 2:** Number of rovings per pass and total passes for final plates layup.

Layer:		1	2	3	4	5
Long One-way span A	Rovings	12	12	–	10	8
	Passes	2	1	–	2	2
Two-way span	Rovings	12	12	–	8	8
	Passes	1	1	–	1	1
Long One-way span B	Rovings	12	12	12	8	8
	Passes	2	1	1	1	1

### Final structural results

**Fig. 20:**
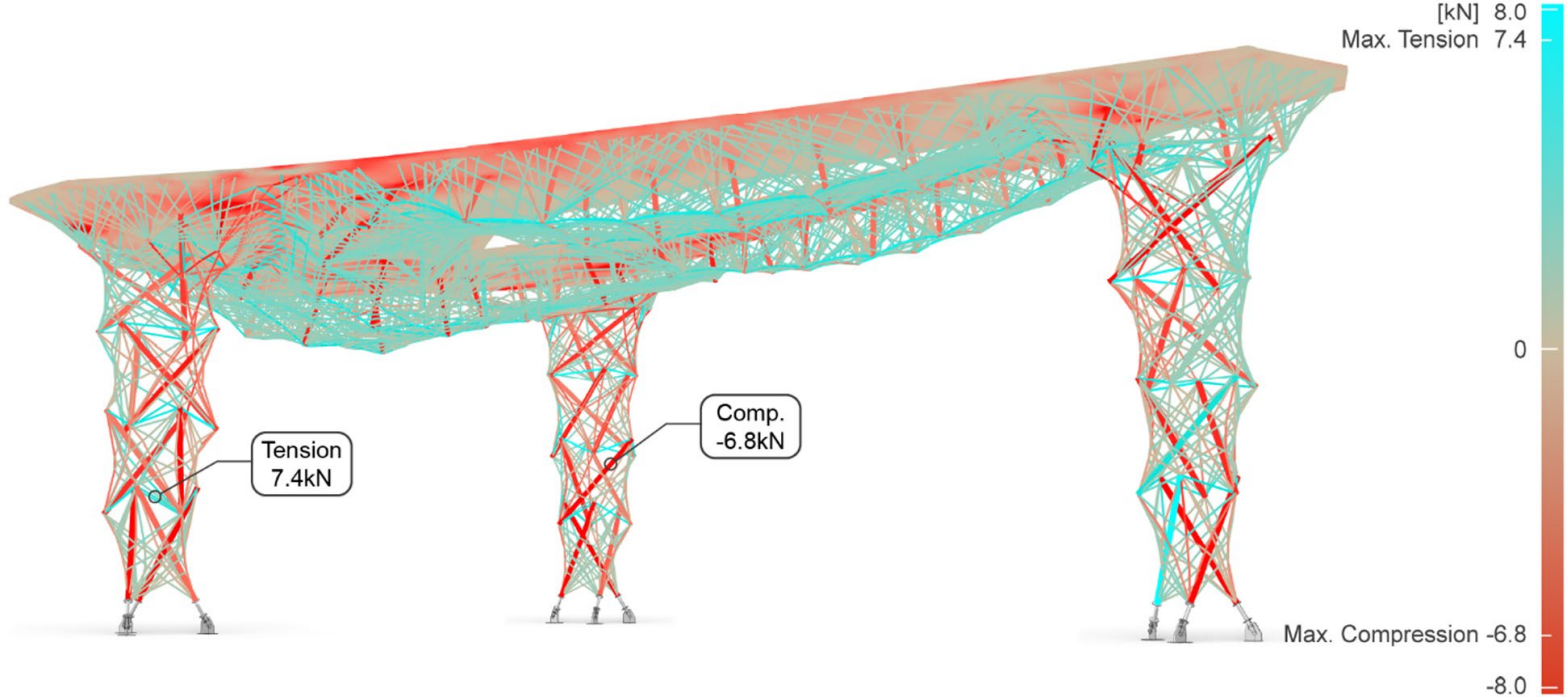
Global FE model and the internal force distribution under the governing load combination (1.35 G + 1.5 W_downward_). Callouts indicate the maximum tension (on a fiber bundle) and the maximum compression (on a timber strut).

The hybrid structure is simulated with FE beam models under the critical load combination of self-weight and downward winding pressure according to Eurocode^60^. The designed rovings per pass and number of passes are transferred into fiber bundle thickness represented by circular rod section diameters. The columns and plates are simulated separately and then assembled into a global simulation to validate the whole system. An overall result of the internal force distribution is shown in Fig. 20. The envelop load combination includes gravity and downward wind, resulting in a mixed compression and tension distribution in the fibers. In the two back columns, vertical fibers are under compression, along with the timber struts, while the horizontal, ring fibers are in tension. Meanwhile, in the frontal column, the main zigzag fiber pattern, designed to carry tensile loads, is predominantly in tension, and part of the vertical fibers also engages in tension, revealing the column’s reaction under lateral loading.

The compression forces concentrate mostly on the timber top slabs and struts in the plates, while in the columns, both timber struts and fiber take compression. For plates, tensile forces are distributed through the parallel fiber spines. In the columns, most tensile forces fall on the horizontal fibers embedded in the *Column Truss 2* layer, which acts as a lateral bracing to hold the vertical struts and fibers from buckling out. Maximum internal forces are all found in the back columns. The maximum tensile force in the fiber bundles is 7.4 kN, and the maximum compression in the fiber is 6.6 kN, and in timber, 6.8 kN. The maximum tensile force value in the plate fiber is 3.5 kN, while the maximum compression in the plate timber struts is 2 kN. In the meantime, support reaction forces are collected for the foundation joint design.

Under the service limit state of combined dead load and wind load, the maximum deformation from the global simulation reaches 20.8 mm around the aperture along the main span. For the overall suspended span of 5.6 m, this maximum deformation equals roughly to L/270. Detailed local fiber buckling was further checked for the columns in a separate simulation model. A pushover force representing lateral wind is additionally considered in the column simulation. Under this condition, higher compression stress appears in the timber struts and fiber bundles compared to what is observed in the global model. The structural check of the columns examines the critical local buckling modes and ensures that the critical buckling factors remain greater than 1.0 and that the overall structural integrity is not governed by local stability failures.

Aiming to better understand the hybrid system’s performance, it was benchmarked against a full timber structure. If the structural system were composed solely of the 42 mm three-layer timber plate (Dreischichtplatte), without the underlying fiber reinforcement, the total deformation of the system would increase to approximately 110 mm. Furthermore, the study compares the performance of the proposed timber-fiber hybrid structure with that of a conventional cross-laminated timber (CLT) slab system, point-supported by timber columns. Under identical design loads and boundary conditions, the fiber-timber hybrid pavilion achieves the same maximum deformation as a conventional CLT slab while reducing the total structural weight by 48%. Whereas the hybrid system weighs 1063.5 kg, the equivalent CLT solution would require a 130 mm thick slab (30-20-30-20-30 mm), resulting in a total weight of 1938.7 kg, an increase of 875.2 kg.

### Fabrication

**Fig. 21:**
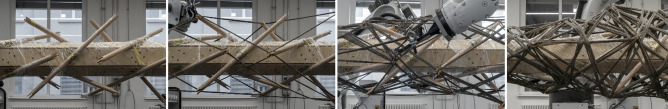
Fiber syntax buildup during pre-fabrication of column component. © ITECH/ICD/ITKE University of Stuttgart.

**Fig. 22:**
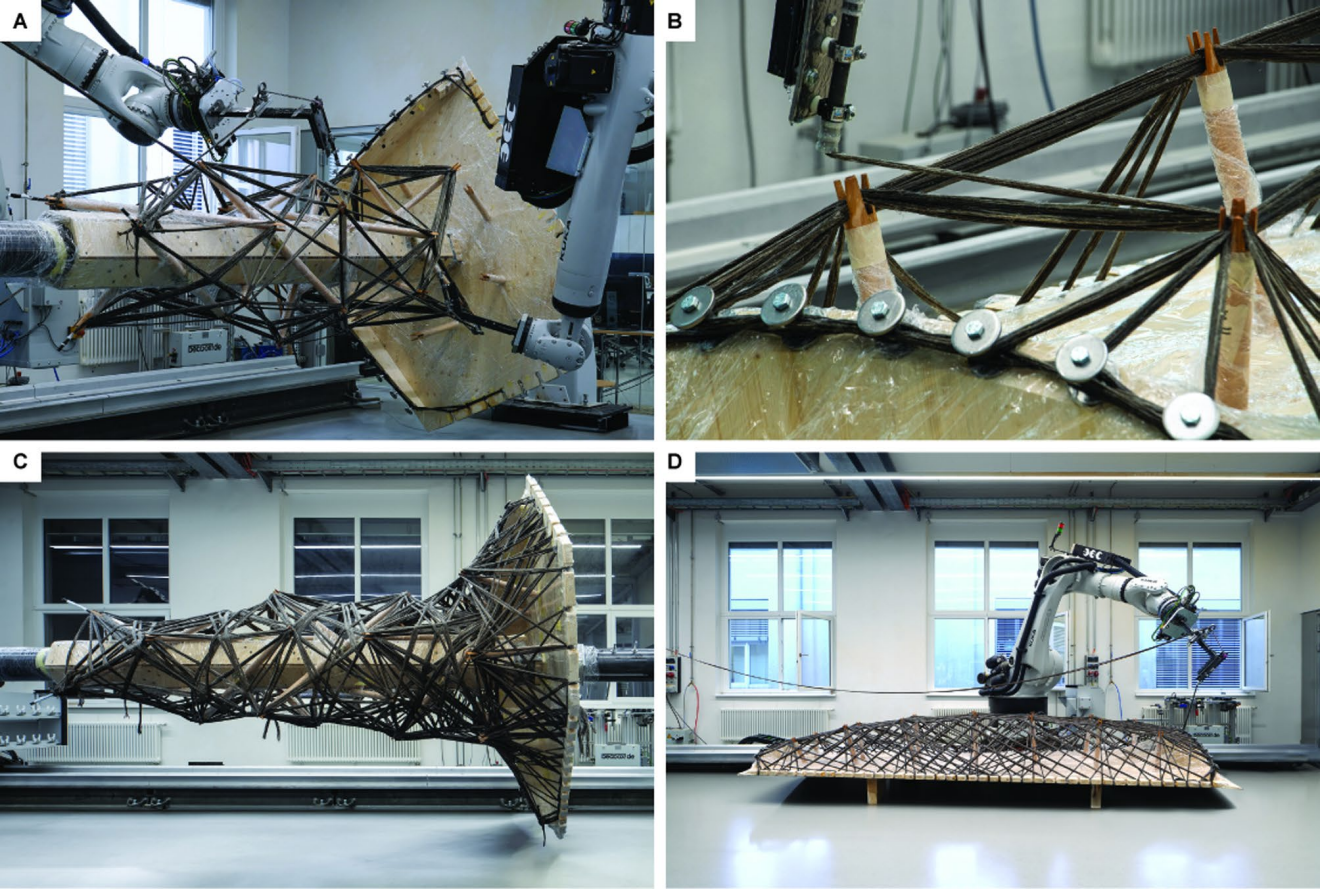
Pre-fabrication of hybrid column and plate components: (**A**) column fabrication with dual-robotic winding strategy; (**B**) strut winding during plate fabrication; (**C**) finished column component; (**D**) finished plate component. © ITECH/ICD/ITKE University of Stuttgart.

The robotic winding of the building components occurred in-house at the Computational Construction Laboratory (CCLab) (Figs. 21, 22). Each column took approximately 14 hours to wind, whereas the plates required an average of 8 hours each. The sequential formation of the column’s fiber layup can be seen in Fig. 21, with the timber struts temporarily secured into the wooden planks, and the consecutive layers building up until the final hybrid geometry was formed. Before each winding session, the placed component needs to be surveyed to calibrate the digital fabrication model. Afterward, updated robot codes that reflect the physical placement of the winding anchors can be quickly generated. Due to the precise CNC milling of the timber plates, only a few of the finger joints need to be surveyed. The column components were fabricated first (Fig. 22A–C). Afterward, the robot cell was reconfigured to a single-robot winding to enable the fabrication of the plate components (Fig. 22B–D). The winding process itself required a minimum of three personnel: one individual to operate the robot, another to monitor the resin bath and manage fiber spools, and a third person to assist with fiber placement and address any arising issues. After each winding session, the components underwent curing in a customized oven. Once the fibers were cured, the brackets securing the column struts to the positioner were removed, allowing the hybrid column to be manually slid out of the core.

After fabrication, a full-scale test assembly of the pavilion was conducted upside-down in the CCLab to assess fabrication tolerances. This process also allowed for the winding of the detachable fiber stitches in place (Fig. 14B). After removal of the temporary frames, additional sleeves and washers could be bolted to the fiber-fiber connection edges and directly wound onto to manufacture the connection pieces. This was done manually, as the test assembly was done outside of the robot’s working area. After hardening, the connection pieces could be removed and fully cured in the oven. Finally, the timber elements were weatherproofed before transporting all components to the site.

### Assembly

**Fig. 23:**
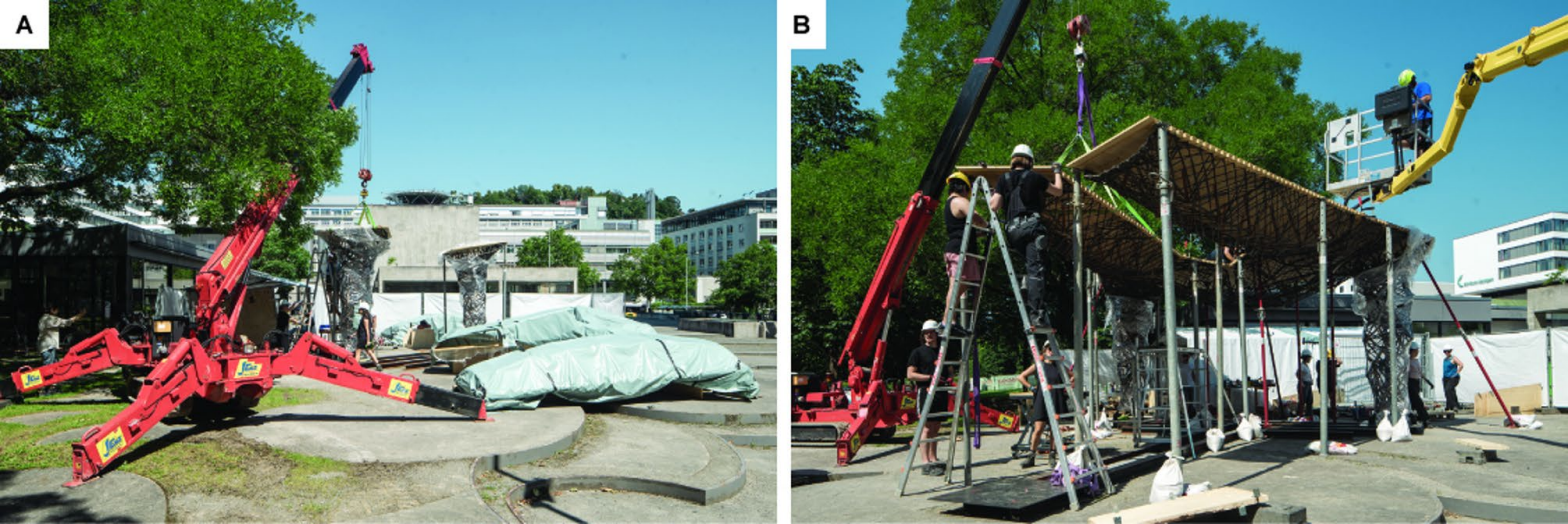
On-site assembly of the ITECH Research Pavilion 2024: (**A**) Placement of back columns using a spider crane, (**B**) assembly of plate components with scaffolding. © ITECH/ICD/ITKE University of Stuttgart.

The assembly process utilized a mini spider crane to lift the components and a telescopic platform to facilitate vertical construction access (Fig. 23A). Temporary steel supports provided scaffolding for the plate components until the third column was placed and connected (Fig. 23B).

The main structure was assembled over the course of two days, with an additional week required to finalize the roof membrane and foundation. The final pavilion covers an area of 45 square meters and weighs 1063.5 kg (23.63 kg/m^2^). It features main spans measuring 5 and 7.5 m. The construction utilized 41.5 km of flax fiber rovings, 1.75 m^3^ of 42 mm thick three-layer softwood timber plates, and 0.096 m^3^ of hardwood struts.

**Fig. 24:**
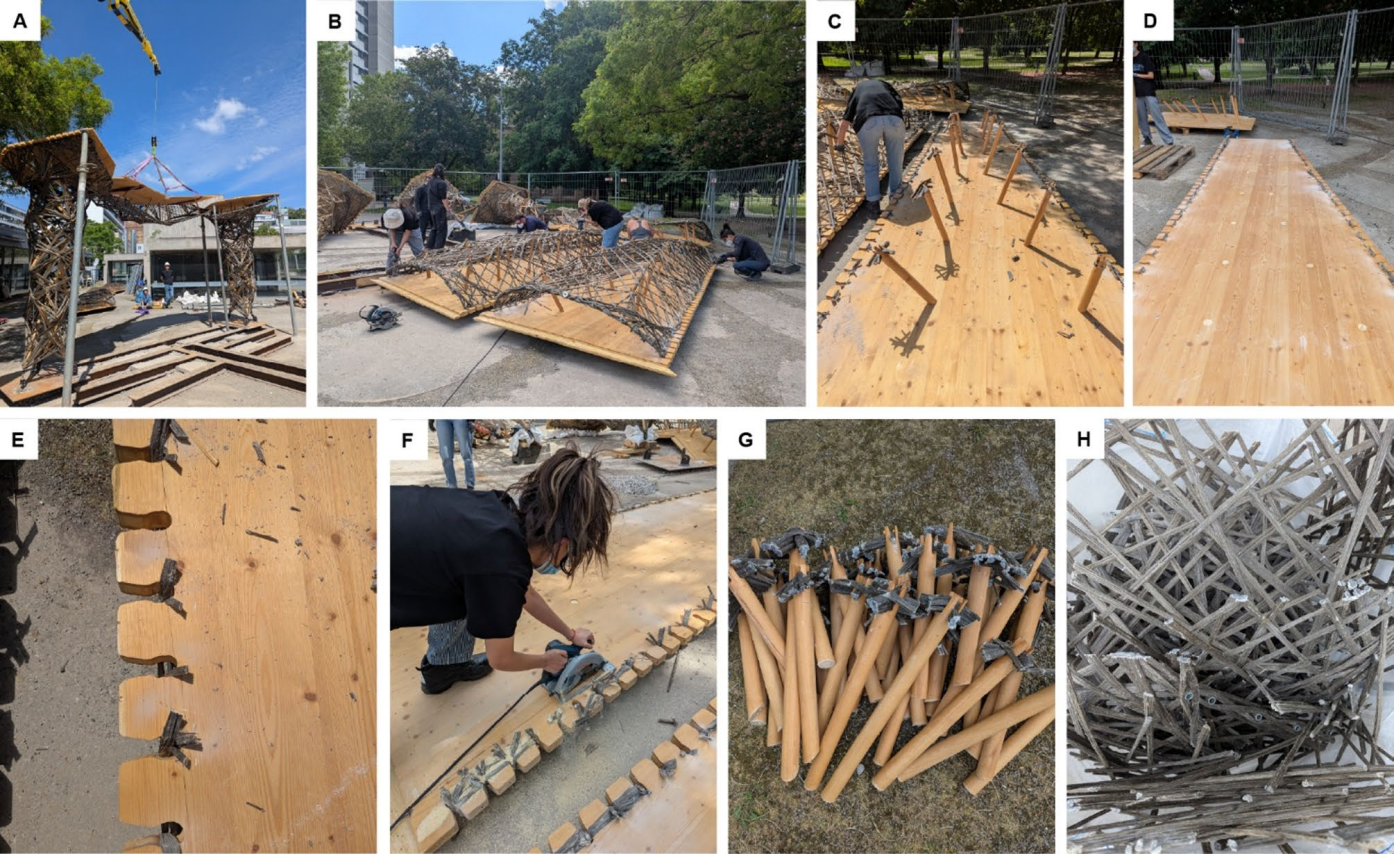
Disassembly Process. (**A**) Structural components were disconnected and lifted using a spider crane, (**B**) Interfaces between timber and NFPC were cut, (**C**) NFPC mesh was removed, (**D**) Screws were removed, and timber struts were detached from plates, (**E**) Detail of interface between NFPC and timber plates after being cut, (**F**) Removal of the timber plates edges (**G**) Timber struts after disassembly, with NFPC remnants removed to enable reuse, (**H**) Collected NFPC mesh offcuts prepared for repurposing.

## Discussion

Integrating natural materials into hybrid systems offers a promising strategy for addressing environmental challenges in construction. A balanced and responsible approach to natural resource use is essential to mitigating the sector’s global impact. NFPC and timber, when strategically combined, complement each other’s properties, enhancing structural performance and enabling morphologies unattainable with a single material alone. The presented NFPC-timber system exemplifies a high degree of interdependency, where both materials play crucial roles in structural, architectural, and fabrication domains. The lightweight, dematerialized nature of the structure highlights its potential as an alternative to traditional building materials, reducing resource usage while maintaining structural performance.

This research explores how computational design and robotic fabrication can be applied to the development of bio-based hybrid systems, with a focus on material efficiency and structural performance. While the project builds on established methods in coreless filament winding and bio-based composites, it introduces a technically significant refinement in the integration of these systems for architectural applications. Rather than proposing a singular material solution, the project investigates hybridization as a means to diversify material use and reduce reliance on individual resources. In this context, the developed methods were implemented and tested through the design, fabrication, and assembly of a research demonstrator. The following subsections discuss the main contributions of this work, along with its limitations and remaining challenges.

### Timber as embedded frame

The integration of an embedded timber frame demonstrates the potential of incorporating a multifunctional element that contributes to both structural integrity and the fabrication process. This approach reduces the reliance on custom-manufactured steel components, which are typically assembled into winding frames but discarded after production, generating significant material waste. In contrast, embedded timber frames remain within the component after curing, becoming an integral part of the structural system.

Despite its benefits, this study also reveals some challenges associated with the use of embedded timber frames. One key issue is the reduced stiffness of the frame during fabrication, which requires design and fabrication strategies to provide higher tolerances and path planning adjustments due to the shifting of strut anchor points caused by frame deformation. Another important aspect concerned the potential irreversibility of the connections between timber and fibers. Although the use of milled timber geometries as integrated winding anchors initially suggested limited separability after curing, the disassembly process demonstrated that the components could be successfully cut apart, enabling the reuse of both timber and fiber composites (Fig. 24). While the timber plates and struts were redistributed to subsequent projects, the NFPC offcuts were redirected to experimental research involving mycelium-based materials. This demonstrated that the system can still support disassembly and material recovery despite the seemingly permanent connections. The embedded connections between timber and fiber are limited to concentrated contact points, allowing the two materials to be easily separated by cutting at those interfaces. Timber elements, steel parts, and gravel from the foundation were also distributed and repurposed into new projects. Future work should further develop strategies for natural fiber composite recycling and full structural reuse, reinforcing circular economy principles. Furthermore, the interface between fiber and timber holds significant potential for future research. Detachable connections, such as the fiber stitch presented in this paper, leverage the lightness and customizable nature of NFPC while facilitating on-site assembly and easy disassembly. The highly experimental and iterative process used to develop these connections opens a promising avenue for deeper investigation. Finding a compromise between material integration and easy disassembly could improve circular design-to-disassembly processes even further.

### Limitations and opportunities of dual-robotic winding

In evaluating the strengths and challenges of the presented fabrication process, several notable contributions to the field of robotic filament winding are identified. Compared to previously used steel winding frames, the use of CNC-milled finger joints significantly reduces the need for time-consuming surveying of winding anchors, as the geometry precisely matches the CAD model. The cooperative winding by two robots facilitates the use of slender timber struts, enhancing overall structural efficiency. However, this process also introduces challenges, such as difficulties in winding onto the struts due to the dynamic addition of tension with each successive layer.

Challenges to fully automate the fabrication process include issues such as up-winding, where fiber bundles slip out of the strut grooves if the winding path requires angles that deviate significantly from the cross grooves. This can lead to fibers slipping out of position. Additionally, even though the groove depths are customized to the planned fiber volume, variations in tension and resin viscosity can affect fiber packing, causing some grooves to become overly filled, which leads to issues in later winding stages. A possible solution would be to incorporate higher tolerances in the calculations, followed by trimming any excess timber when necessary. Similarly, accessing the winding points in the inner column layers near the plate becomes increasingly difficult as the process progresses due to potential collisions with previously placed fiber bundles and plate struts. These collisions are challenging to predict when relying solely on robot simulations. However, implementing a more detailed process simulation that accounts for fiber placement during robot motion could potentially address these challenges in the future^58^. Simulating the fiber interaction would also facilitate the automated search for optimal winding sequences^32^, thereby allowing a more comprehensive exploration of the extended fiber syntax design space enabled by dual-robot winding. Moreover, synchronizing the hooking logic for both robots poses a significant challenge, as it requires careful consideration of the varying angles and orientations of the struts to prevent collisions. To the best of our knowledge, no prior work has addressed the parallel filament winding with two fiber bundles, making it a relevant contribution to the field of collaborative robotic fabrication.

### Future directions for hybrid fiber-timber systems

Research on bio-based hybrid architecture, particularly NFPC-timber systems, has demonstrated the potential of these materials for creating lightweight, load-bearing structures that challenge conventional material practices in architecture. Advances in design and fabrication methods are continuously improving, paving the way for their application in permanent structures. However, structurally benchmarking bespoke fibrous systems against conventional building components is challenging, as it requires comparable metrics and comprehensive evaluation methods that can account for uncertainties at both the material and system levels. A full-scale structural test would be required for a comprehensive benchmarking of the system; however, this was beyond the scope of the present study. As a proof of concept, the developed timber–fiber hybrid system was therefore compared to a conventional cross-laminated timber slab with increased plate depth under identical loads and boundary conditions. This simplified comparison highlights a substantial reduction in both timber volume and overall structural weight while achieving comparable structural performance. Specifically, the hybrid system reaches the same maximum deformation as the equivalent CLT solution with a weight reduction of approximately 48%, corresponding to a decrease of 875.2 kg. These results indicate that hybridization can significantly reduce material demand compared to monolithic timber systems. Further investigations should focus on refining the system and validating its performance through full-scale testing, with the aim of further optimizing material efficiency.

The use of a partially bio-based resin demonstrates its applicability in a load-bearing structure. Compared to a conventional epoxy resin from the same producer, the partially bio-based resin achieves a 44.9% reduction in global warming potential, corresponding to 4,220.03 kg CO₂-equivalent for every 1000 kg of resin (LCA Data provided by the resin producer in accordance with ISO 14040/44 conducted in 2022). However, only 51% of the resin’s molecular content is derived from plant-based sources. When accounting for the fossil-based hardener, the resulting matrix system contains a total of 39.2% bio-based content, which limits its overall ecological potential. Further exploration of fully bio-based resin alternatives could enhance environmental performance and improve end-of-life options. Ongoing investigation by the authors^61^ into the LCA of the hybrid system used in the Hybrid Flax Pavilion indicates that the hybrid roof structure could substantially reduce timber use (53,8%) and embodied carbon emissions (34% compared to the German building average), while also revealing the environmental drawbacks of steel connectors and fully petrochemical resins. Future studies could perform a full life cycle assessment of the pavilion and benchmark it against other systems, providing a quantitative understanding of the environmental benefits of combining timber and flax. Additionally, manufacturing scalability and long-term durability are key factors essential for real-world adoption. Ongoing research^62^addresses these concerns by investigating the long-term performance and flammability of NFPC-timber composites in permanent structural applications. Further research is required to generate comprehensive data on the mechanical performance of NFPC-timber hybrids under varying environmental conditions and to enhance the fire resistance of the composite material, increasing the practical applicability and regulatory acceptance of this structural system.

Beyond the pavilion-scale demonstrator, the integration of both single-span and multi-span hybrid components demonstrates the system’s flexibility in adapting to different scenarios regarding scalability and architectural applicability. The suitability for multi-story applications should be considered in future work, taking into consideration façade and building system integration. From a fabrication perspective, further research is required to assess scalability, particularly regarding the use of timber as an embedded frame and the management of fabrication tolerances at larger production scales. Nevertheless, the use of integrated tooling, combined with cooperative robotic workflows, and the modular nature of the designed system indicate a potential pathway toward economic scalability, particularly for extending existing building stock, where lightweight structures with material efficiency, adaptability, and architectural differentiation are prioritized.

## Conclusion

This project introduces a new approach to hybrid bio-based construction by integrating natural fibers and timber into a load-bearing lightweight system, building on recent advances in the field. The findings demonstrate how computational design and robotic fabrication methods enable enhanced material efficiency via strategic placement of timber and NFPC, reduce dependency on single resources, and introduce new possibilities for architectural applications. The embedded timber frame strategy, in particular, demonstrated potential in reducing fabrication waste and enhancing structural efficiency, although challenges remain in ensuring fabrication precision. While the research successfully advances the use of bio-based materials, further reducing reliance on petroleum-based resins will be essential for improving full circularity. The dual-robot winding technique broadens the design possibilities of fiber structures, providing new solutions for the fabrication of integrated hybrid systems. Addressing these limitations will be key to expanding the applicability of hybrid NFPC-timber systems in both temporary and permanent structures. Ultimately, this project contributes to the ongoing discourse on bio-based architecture by demonstrating how hybrid material systems, combined with advanced digital methods, can redefine bio-based building systems. As future research continues to explore material innovations, fabrication techniques, and ecological design principles, bio-based hybrids have the potential to contribute to a more sustainable and resilient built environment.

## Data Availability

Data can be made available upon reasonable request from the corresponding author.
